# Mechanical Energy Sensing and Harvesting in Micromachined Polymer-Based Piezoelectric Transducers for Fully Implanted Hearing Systems: A Review

**DOI:** 10.3390/polym13142276

**Published:** 2021-07-12

**Authors:** Rhonira Latif, Mimiwaty Mohd Noor, Jumril Yunas, Azrul Azlan Hamzah

**Affiliations:** Institute of Microengineering and Nanoelectronics, Universiti Kebangsaan Malaysia, Bangi 43600, Malaysia; mimiwaty@ukm.edu.my (M.M.N.); jumrilyunas@ukm.edu.my (J.Y.); azlanhamzah@ukm.edu.my (A.A.H.)

**Keywords:** hearing aids, implantable microphone, MEMS sensor, piezoelectric-polymers, energy harvester

## Abstract

The paper presents a comprehensive review of mechanical energy harvesters and microphone sensors for totally implanted hearing systems. The studies on hearing mechanisms, hearing losses and hearing solutions are first introduced to bring to light the necessity of creating and integrating the in vivo energy harvester and implantable microphone into a single chip. The in vivo energy harvester can continuously harness energy from the biomechanical motion of the internal organs. The implantable microphone executes mechanoelectrical transduction, and an array of such structures can filter sound frequency directly without an analogue-to-digital converter. The revision of the available transduction mechanisms, device configuration structures and piezoelectric material characteristics reveals the advantage of adopting the polymer-based piezoelectric transducers. A dual function of sensing the sound signal and simultaneously harvesting vibration energy to power up its system can be attained from a single transducer. Advanced process technology incorporates polymers into piezoelectric materials, initiating the invention of a self-powered and flexible transducer that is compatible with the human body, magnetic resonance imaging system (MRI) and the standard complementary metal-oxide-semiconductor (CMOS) processes. The polymer-based piezoelectric is a promising material that satisfies many of the requirements for obtaining high performance implantable microphones and in vivo piezoelectric energy harvesters.

## 1. Introduction

The ear and brain work together in the hearing process. Sound energy in the audible frequency range (20 Hz–20 kHz) propagates through air medium as an acoustical mechanical wave which enters from the outer ear towards the middle ear to vibrate the eardrum. In a healthy cochlea, the eardrum vibrates the small bones called the ossicles (malleus, incus and stapes) and the sound vibrations travel to the cochlea which resides in the inner ear. The basilar membrane (BM) in the cochlea mechanically filters sound while the hair cells turn BM vibrations into electrical nerve impulses. In this mechanoelectrical transduction, BM vibrations displace hair cells (stereocilia) resulting in the gating of ion channels and generating bioelectrical potentials. The auditory nerves are stimulated and the brain would interpret the information received from the generated nerve impulses.

There are three main types of hearing loss that can either affect one or both ears. Conductive hearing loss occurs when the outer ear and middle ear could not become the transmission mediums to conduct sound properly. People with severe outer or middle ear malformation and chronic ear infection may experience conductive hearing loss. The sensorineural hearing loss happens due to missing or damaged hair cells in the inner ear that lead to frequency selectivity and sensitivity disorders. Mixed hearing loss is the combination of both conductive and sensorineural hearing losses, a result associated with issues related to both outer/middle ear and inner ear. Different types of hearing devices like cochlear implant (CI) and middle ear implant (MEI) have been developed to cater to different types of hearing problems. Hearing device usage significantly improves the social, psychological, emotional and physical aspects of life for hearing impaired persons.

**Figure 1:**
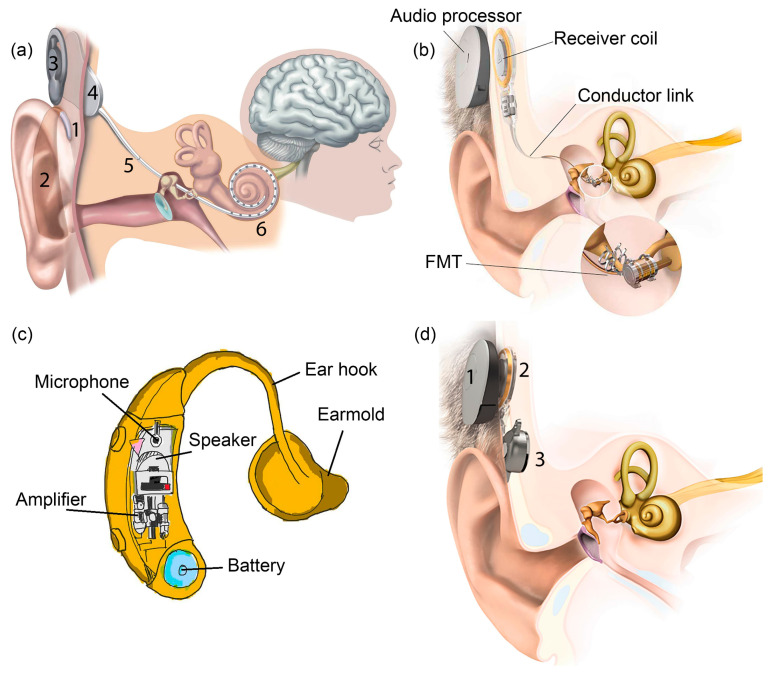
The schematic of (**a**) cochlear implant (CI) [1], (**b**) middle ear implant (MEI), photo credit: MED-EL [3], (**c**) hearing aid and (**d**) bone-anchored hearing aid (BAHA), photo credit: MED-EL [9].

CI is the most successful neural prosthesis to date with a mature technology in biomedical device implantation. It mainly treats patients with severe to profound sensorineural hearing loss. Figure 1a displays the externally worn audio processor (2) with a battery pack behind the ear and a microphone (1) hooked onto the ear [1]. The microphone transduces acoustic waves from the environment into electrical signals before feeding them into the processor. Bank of filters in the processor decompose the electrical sound signals digitally into different frequency bands/components and implement certain specific processing strategies to extract the right features from the detected sound. The signals are encoded into radio frequency (RF) electrical signals. RF transmitter coil (3) is positioned in place and aligned to the RF receiver coil (4) by a magnet. The coded signals are transmitted to the receiver coil implanted under the skin from the transmitter coil via transcutaneous magnetic induction mechanism. The hermetically sealed implanted stimulator circuit (4) derives power from the RF signal and translates the coded signals into electrical pulses. A conductor link (5) threaded into the cochlea and the multichannel stimulation electrodes (6) at the end of the conductor link connect to the hearing nerves. The electrical pulses are transmitted from the stimulator to the stimulation electrode array inserted in the cochlea via the conductor link and directly stimulate the auditory nerves. All of these processes are supported by a battery, supplying each component of the CI with power [1,2].

MEI has a similar arrangement like CI with the extracorporeal audio processor and surgically implanted receiver coil under the skin (Figure 1b) [3]. Magnetic attraction holds the audio processor directly over the implant. A microphone detects sound and converts it into electrical signals. The processor processes the electrical signals and transmits them to the implant before being relayed to the floating mass transducer (FMT) via a conductor link [4]. FMT converts the electrical signals into mechanical vibrations that set the middle ear structures into motion and subsequently transfer the vibrations to the cochlea. At this stage, the cochlea may proceed with its natural functionalities by which the BM mechanically filters the sound vibration and the hair cells transform the vibration into bioelectrical potentials. There is a variety of types of MEI based on how FMT is attached in the middle ear. FMT can be clamped to the incus of the ossicular chain or inserted into the round window of the cochlea [5]. The working operation of MEI is independent of skull growth and thus suitable to be implanted in children [6]. MEI can aid people with a conductive, mixed or mild to severe sensorineural hearing loss.

Figure 1c shows a hearing aid which is basically an acoustic amplifier [7]. The detected sound by microphone is digitally amplified by the audio processor. The increased sound volume is then transmitted to the ear canal via a customised earmold worn in the ear. The loudspeaker converts back the electrical signals into sound signals and delivers them to the ear drum in the middle ear. The ossicles and cochlea will naturally process the sound signals. Deaf patients with less than 70–80 dB of sensorineural hearing loss can use a hearing aid [8]. 

Bone-anchored hearing aid (BAHA) is a hearing system based on bone conduction method, specifically designed for people with damaged outer or middle ear. It utilises the skull’s bones to conduct sound into the inner ear, facilitating hearing for people with a conductive or mixed hearing loss condition. In Figure 1d, the externally worn microphone and audio processor (1) pick up sound and convert it into electrical signals [9]. These signals are transmitted to the RF receiver coil (2) which has been surgically positioned transcutaneously under the skin around the temporal bone. An actuator/vibrator (3) transduces the electrical signals into mechanical vibrations and the signals are transmitted to the cochlea through the skull. The vibrations make the cochlea fluid move and generate nerve impulses [10,11]. Adhesive bone conduction system is a newly developed non-surgical device where the users can simply stick an adhesive adapter onto the skin behind the ear [12,13]. The audio processor clicks onto the adapter where it picks up sound and vibrates the adapter. The adapter then gently vibrates the bone behind the ear. The bone conducts the vibrations towards the cochlea. The system comfortably avoids pressure onto the skin and can be worn unnoticeably behind the ear.

A summary detail on the commercially available hearing systems is tabulated in Table 1. HiRes Ultra 3D by Advanced Bionics is a CI system with 16 stimulation electrodes that can deliver up to 120 spectral bands of sound [14]. Greater spectral resolution improves speech perception in noise and music appreciation. Nucleus^®^Systems by Cochlear Americas possesses 22 stimulation electrodes that provide access to the full spectrum of sound and optimal hearing coverage. The recipients’ number of Nucleus^®^Systems has grown to more than 400,000 worldwide [15]. It is the first CI with removable magnet, allowing users to undergo magnetic resonance imaging (MRI) brain scans. Vibrant Soundbridge by MED-EL is a semi-implantable MEI that implemented mechanical stimulation using electromagnetically driven transducers [16]. Fully implantable piezoelectric-based MEI, Envoy Esteem by Envoy Medical is widely used in the United States and Europe [17]. Evoke by Widex is the first brand that incorporates machine learning into the hearing aid [18]. The intelligent hearing aid is capable of learning from different situations and user’s inputs in order to evolve its own functions, so that high quality sound can be heard. More by Oticon is a recently developed hearing aid with internet-enabled and Bluetooth-enabled functions [19]. Baha^®^Attract is a BAHA system manufactured by Cochlear corporation [11]. The vibrator is magnetically retained externally with the audio processor to the bone conductor. Soft pad is used to distribute pressure over the area of skin contact for magnetic attachment [13]. On the other hand, BONEBRIDGE by MED-EL is a different BAHA system that has an implanted vibrator in the mastoid area to directly connect to the skull [13,20]. The exterior processor is quite light due to the exclusion of the vibrator’s weight. 

Even though there are huge technological improvements in these hearing devices with tremendous technical and clinical success, the number of hearing device adoption rate has not increased [21]. More than 75% of hearing impaired population did not benefit from the advanced hearing devices technology, making hearing loss as the largest chronic sensory condition that remains untreated [16]. The main reason for rejection of becoming a hearing device user is the stigma of wearing the device itself. Patients are concerned with society’s perspectives on the devices’ cosmetic appearance and wish for their deafness to be hidden. In addition, there are risks for the device to be damaged as the exterior parts can be subjected to trauma, humidity or dirty conditions. The magnetic fixation of the RF transceiver coils can cause problems in the skin tissue due to continuous pressure exercised by the antennas. Other limitations include the restriction from enjoying physical activities like swimming due to water exposure or extreme sports due to perspiration. Simple daily routines like showering and sleeping require the users to temporarily remove the external part [6,16,21,22]. Many users desire the hearing devices to work without the exterior components on or off the ear with 24/7 operation. It has become a quest for researchers to invent a totally implantable hearing system that would make the device invisible. 

Only a few fully implanted MEI systems have been reported and the semi-implanted MEIs have been found to have less performance problems compared to the fully implantable ones [23]. Hearing aid is not an implant but an externally or internally worn device. Behind the ear (BTE) and receiver in the canal (RIC) are body-worn hearing aids where the loudspeaker is located behind the ear and in the earmold, respectively. In the ear (ITE) fits in the outer ear bowl while a completely in the canal (CIC) hearing aid is placed deep in the ear canal, barely visible. An invisible in canal (IIC) hearing aid is positioned completely inside the canal leaving no visible trace. It is not possible to have a fully implanted system for BAHA while for CI, many are still in the research phase and not yet commercialised. Recently, MED-EL announced the very first surgery of a fully implanted CI in Europe and will receive its market approval in the next several years [24]. In this paper, we will look closely at the efforts done towards achieving the fully implanted systems and discuss the key challenges and limitations that impede the process of attaining one. The battery, microphone and processor from the externally worn compartment of the hearing systems are thought to be the three main issues that need to be addressed and discussed thoroughly. Therefore, this review article is structured into two sections: energy harvester and implantable microphone. A review of the low-powered speech processing strategies and the programmable processor circuit implementation for the hearing system’s processor unit will not be presented in this paper. For each section of the microphone sensor and energy harvester, comprehensive background information on the electromechanical energy sensing/harvesting and the available transduction mechanisms are discussed in depth. None of the recent reviews compile both sensor and energy harvester in one literature which focuses specifically on hearing system application.

Microfabrication technology offers device size reduction and compatibility with the silicon processes, allowing the microphone and energy harvester to easily integrate with the digital signal processing circuitries in one small chip that can fit into the ear. In the end, the choice of materials to be implemented is of utmost important. The microphone and energy harvester for fully implanted hearing systems are desired to operate efficiently, pose low health risks and be compatible with complementary metal-oxide-semiconductor (CMOS) processes. In vivo energy harvesters especially need to be flexible and stretchable. The combination of piezoelectric materials and polymers into a nanocomposite or a multi-layered thin film appeals as a promising structure for both energy harvesters and microphones. Thus, in this paper, we will mainly focus on the sensing and energy harvesting of polymer-based piezoelectric transducers for totally implanted hearing systems. The design principles of the piezoelectric/polymeric devices are carefully examined, which include the materials development, configuration structure and fabrication, operation modes, packaging strategies and considerations of biocompatibility. In order to clarify the advantages of piezoelectric/polymeric device structure in hearing implants and to further evaluate their potentials, detailed reviews on a range of piezoelectric material including ceramics, polymers and composites and a range transduction mechanism like electromagnetic induction, electrostatic or triboelectric is also presented. All in all, we have found that a piezoelectric/polymer combination material satisfies many of the requirements and future opportunities include the development of a biocompatible, electromagnetic-compatible, CMOS-compatible fully implanted hearing device system in the ear cavity. 

## 2. Energy Harvester for Totally Implanted Hearing Device System

Generally, an implanted medical device is used for sensing or stimulating by which it serves as a diagnostic tool or is used for treatment practices. A patient who is suffering from neural system disorder uses implantable stimulators [25], Parkinson’s symptoms can be eased via deep brain stimulators [26] and a hearing impaired person uses a cochlear implant for hearing restoration [27]. These medical implants, including cardiac pacemakers, retinal implants and infusion pumps, are considered small-sized and low-powered electronic devices. The electrochemical battery to power up these devices needs periodic recharging or has a limited lifetime that requires replacement. The implanted lithium battery for a pacemaker, neurostimulator and insulin infusion pump needs to be replaced every ~6.5 years, ~4 years and ~2 years on average, respectively [28,29]. The implanted battery for a MEI can stay for ~6 years while an external worn battery has to be charged every week [4,30]. Higher energy usage will reduce the lifespan of an implant’s battery and its small energy storage requires frequent charging. The removal and replacement of an implanted battery requires surgery that could pose risks to the well-being and health of the patients. The risks of infection, bleeding, inflammation, long healing process and additional cost have called for the development of self-powered medical implants to reduce these physical, psychological and financial burdens on users. Self-sustainable energy generation may perhaps ensure the longevity of the implanted medical devices.

### 2.1. Energy Harvesting Technologies for Implanted Medical Devices

Energy harvesting can extend the working life of electronics. Battery contributes an unwanted weight and volume in a biomedical system. A battery that can last longer presages an increase of size and weight of the implanted devices. Therefore, an energy harvester is essential in the manufacturing of medical implants. There are various accessible energy sources like solar, heat, electromagnetic radiation, ultrasonic waves, fluid flow and vibration which could be used to generate electrical signals. From these sources, there are different types of transduction mechanisms that can be implemented in implanted biomedical devices like piezoelectricity (mechanical strain), electromagnetism (magnetic induction), electrostaticity (capacitance), thermoelectricity (temperature gradient), triboelectricity (frictional contact and electrostaticity) and pyroelectricity (thermal fluctuation). Two energy harvesting concepts are coined, either from the environmental source or human body. 

**Figure 2:**
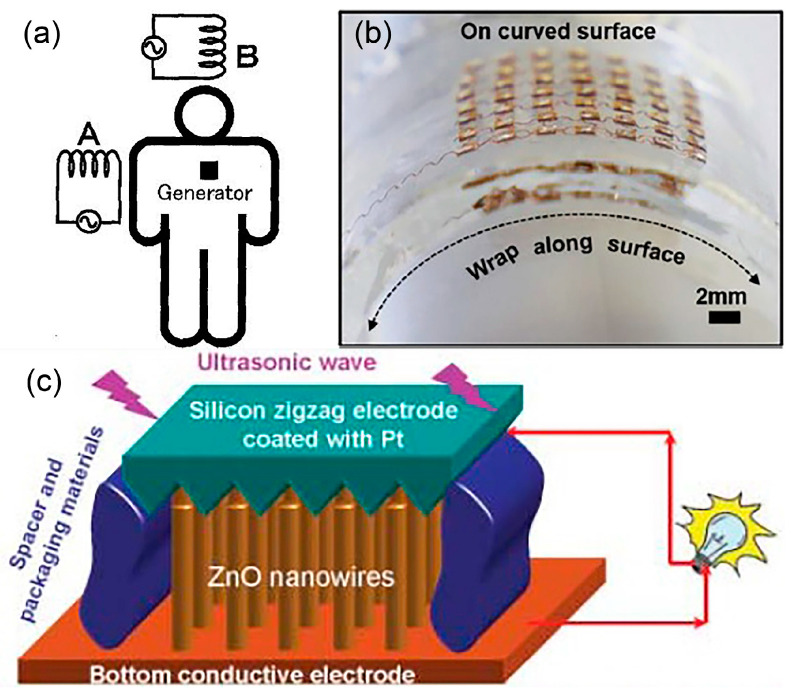
(**a**) Electric power generating system for implanted medical devices is proposed by positioning a human body in a rotating magnetic field. © 1999 IEEE. Reprinted, with permission, from [31]. (**b**) Optical image of the flexible piezoelectric transducers array for ultrasonic energy harvesting. Reprinted from [32], Copyright (2019), with permission from Elsevier. (**c**) A schematic diagram of a nanowire piezoelectric nanogenerator driven by an ultrasonic wave [33].

Energy sources from the outside of a human body can be utilised by the implanted energy harvesters to generate electrical energy. Wireless energy transfer is a reliable and convenient through-skin energy transmission method to charge up the implanted biomedical device or energy storage device without additional surgery [25]. Reported here are few examples of implantable wireless energy harvesters that a hearing device could adopt for its own fully implanted system. In [31], electromagnetic induction has been used to power up a pacemaker. A human body is proposed to be placed in a low frequency rotating magnetic field that triggers the implanted microgenerator (Figure 2a). Two phase excitation coils are set up outside of a human body while the microgenerator, metal magnet and high-ratio gear are implanted inside. A rotating magnetic field is applied onto the implanted magnet causing it to rotate. High-ratio gear increases magnet rotation and drives the microgenerator to generate high voltage. The system produces small eddy current due to low frequency excitation and thus it is safe to be implemented in a human. They estimate that 4 W of power is needed to be generated from the system to fully charge a pacemaker for 3 h.

In [32], the wireless energy transfer employs an acoustic energy to supply power for the implanted medical devices. A flexible piezoelectric transducers array is developed (Figure 2b) for ultrasonic energy harvesting. It generates continuous ~2 peak-to-peak voltage (Vpp) output and ~4 µA current under ultrasonic excitation, when implanted on both planar and curved surfaces. The developed flexible device demonstrated auspicious performance with weak attenuation on curved surfaces. The proposed design solved the bulky and rigid issues of other ultrasonic energy harvesters that restrict them from attaching to soft and curvy surfaces. Wang et al. have also driven an implanted piezoelectric-based nanogenerator device using ultrasound that can produce continuous direct current (DC) output for an implanted device [33]. In Figure 2c, the nanogenerator is made of vertically aligned zinc oxide (ZnO) nanowires grown on a polymer substrate and covered by a zigzag electrode. 

Semi-implanted hearing systems, however, have already adopted the wireless energy transfer method via inductive coupling based on the electromagnetic field between the RF transmitter and receiver coils. The coils have a dual function of delivering power and auditory information, simultaneously. The external battery with rechargeable characteristics continuously supplies power to the internal implant coil wirelessly. For a cochlear implant, a small implant coil is adequate with a power requirement of ~20 mW [25]. For a totally implanted hearing system, it is impractical to develop an implanted wireless energy harvester that harvests energy from the external sources. It is a hassle for the user to stay inside the magnetic field or ultrasound wave set up for the hearing device to be continuously powered and operated, unless there is also an implanted energy storage device which will then require periodic recharging [34]. In addition, there might be restrictions or health and safety concerns on the users with implanted electromagnetic-based wireless energy harvester to undergo head MRI. Also, long term magnetic radiation exposure might cause decrease in fertility, muscle stiffness with loss of protein and neuroendocrine system degradation due to change in DNA, while electric fields may cause unnecessary stimulation of muscles, nerves and sensory organs [25]. 

The implanted medical device or energy storage device can be charged internally, directly, continuously and unconsciously by the energy harvested from human body. In vivo implantable energy harvesters are aimed at powering electronic devices embedded inside the human through the body’s thermal gradient or internal biomechanical motion like lung inflation, stomach deformation, cardiac motion, muscle contraction/relaxation or blood flow. A human body at rest consumes ~100 W to maintain internal organs, tissues and cells functioning while it produces ~81 W when asleep and 1630 W during sprint walk [28]. 

Heartbeat vibrations can generate and supply power to pacemakers or sensors that stimulate heart muscles, regulate its contraction and monitor vital signs like pulse rate or blood pressure. In [35,36], fan-folded piezoelectric beam stacked structure uses heartbeat to generate more than 10 µW to power up a lead-free pacemaker. Fan-folded design is chosen in order to utilise three-dimensional space to the energy harvester and the added tip mass and link mass help to reduce the high natural frequency of the energy harvester. Small natural frequency of the device (16.18 Hz) generates more power. Less than 1 µW is needed for a pacemaker and the proposed energy harvester of size 2 cm × 0.5 cm × 1 cm generates sufficient energy for the medical implant. No magnet is incorporated into the proposed device and thus it is MRI compatible. Dong et al. developed a cardiac energy harvester using a porous piezoelectric helical thin film structure that transforms the mechanical energy from a pacemaker’s lead into electrical signals [37]. This strategy allows the battery of a cardiac pacemaker to be charged without the energy harvester coming into contact with the heart and thus interferes with its function. The bioinspired self-wrapping helical configuration of the energy harvester allows flexible integration with the lead of the pacemaker and the bending motion of the energy harvester generates ~0.6 Vpp output. A 10 × 10 array of helical devices wrapping all through the lead is estimated to extend the pacemaker’s battery lifetime by 1.5 years. Kim et al. developed a self-powered cardiac sensor from a flexible piezoelectric transducer that can generate 17.8 V and 1.74 μA from the contraction and relaxation of a porcine heart [38]. In [39], an implantable blood pressure sensor in aorta could monitor high blood pressure using the aorta’s pulsating energy in piezoelectric generator film, producing a maximum output voltage, current and power of 10.3 V, 400 nA and 631 nW, respectively. 

Other than heartbeat, the temperature difference between the skin and body core can be used to generate electricity and charge the battery of a pacemaker. Thermoelectric energy harvester converts temperature gradients into electrical energy and such a small temperature difference could provide high power output of more than 100 µW [28]. Cardiac pacemaker does not require constant battery loading and consumes low energy where 10 µW can sufficiently power up the device [28,35,40]. The biggest consideration of implementing a thermoelectric energy harvester in an implanted medical device is the biocompatibility of the materials used. Bismuth telluride is a common thermoelectric material that is moderately toxic but can be fatal in large quantity [28].

A brain pacemaker is a neurosurgical treatment which stimulates certain area in the brain via implanted electrodes that generate continuous electrical impulses. Deep brain stimulation helps to relieve neurologic and psychiatric disorders like epilepsy and Parkinson’s disease. This neurostimulator consumes more energy compared to a cardiac pacemaker. A flexible piezoelectric thin film energy harvester on plastic substrate by Hwang et al. can be utilised as a self-powered deep brain stimulator using slight movement of human muscles or organs [41]. The fabricated energy harvester yielded a maximum output current and voltage of 0.57 mA and 11 V, respectively. The energy harvester manages to stimulate a specific target area of a mouse brain that instantaneously induces its forearm movement. In [42], Fan et al. have proposed an energy harvesting system utilising the deformation of a piezoelectric material attached onto the human mandible to power up the implanted deep brain stimulator. The piezoelectric energy harvester was mounted onto a synthetic mandible and a novel mandibular loading set up was developed to copy the human mastication forces; 1 Vpp output was measured and the improved device performance could power up a commercial deep brain simulator in the future.

Depending on the stimulation strategies, the average total power consumption for a CI can be around 10 mW. Žák et al. proposed a multidisciplinary ambient energy harvesting system from the combination of thermal gradient, mechanical movement (shocks) and bending movement of neck muscles or arteries in the head area for autonomous powering in a totally implanted CI [43]. For energy harvesting from the mechanical movement, they have suggested the electromagnetic-based electromechanical conversion principle, utilising resonance at the excited mechanical movement/vibration frequency. Although human muscles and skeleton cause a damping effect, it can be neglected for small weight of proof mass. The electromagnetic energy harvester consists of the flexible cantilever structure with seismic mass (permanent magnet) which moves against a fixed printed coil that induces voltage. At 18 Hz, a maximum output voltage and power of ~1.4 Vpp and ~1.8 mW have been simulated, respectively, during walking in the time range of 10 s. As for the thermoelectric energy conversion, the waste heat produced in the region of human head (~15 W) is converted into useful electrical energy via the Seebeck effect. Thermal gradient around 5 °C is desired with the thermal resistance of human tissue between body core and skin surface variying with respect to physical activities, age and health conditions. Thermoelectric module with 100 mm^2^ area is able to provide more than 300 µW of power. Additionally, the electrochemical gradient in the inner ear’s fluid can be used as an energy harvesting source. The difference in ionic concentration between the endolymph and perilymph fluid induces endocochlear potential. In [44], an endoelectronic chip is positioned in the middle ear between two electrodes that connect to the endolymph and perilymph. The energy harvester extracts a minimum of 1.12 nW from the endocochlear potential in guinea pigs continuously for 5 h. Table 2 summarises the various in vivo implantable energy harvesters for powering the implanted medical devices. 

In the search of materials and methods for creating an energy harvester that supports the development of a fully implanted hearing system, the mechanical energy appeals as the most omnipresent energy source available from the ambient environment surrounding the hearing device. The mechanical energy is relevant because it is closely related to the hearing process and can be easily captured before being converted into useful electrical power via mechanoelectrical transduction. The in vivo energy harvesting through human organ motion can supply clean power for the implanted medical device without affecting the organ or its functions. As the electronic device shrinks, so does the energy consumption. Piezoelectric transduction mechanism transduces mechanical vibrations into microwatts and milliwatts of power level and the approach is suited for in vivo energy harvesting in cochlear devices. Therefore, piezoelectric energy harvesting (PEH) can generate sufficient electrical output to meet the requirement of a totally implanted device.

### 2.2. In Vivo Piezoelectric Energy Harvester (PEH)

Piezoelectricity is formed solely based on the inherent polarisation within piezoelectric materials and is not initiated by an external electric field, magnetic field or frictional contact with other materials. Materials which are piezoelectric intrinsically have the aptitude of transforming the mechanical strain into electrical voltage. Piezoelectric energy harvester (PEH) executes a direct piezoelectric effect without a separate voltage source or a permanent magnet, as in the case of electrostatic or electromagnetic energy harvesters, respectively. Piezoelectric transduction is a prominent mechanical energy harvesting mechanism that generates larger power output density compared to electromagnetic, electrostatic and triboelectric transduction-based energy harvesting [29,45]. Microelectromechanical systems (MEMS) transducers are developed using materials and micromachining techniques originated from the microelectronics industry, primarily based on silicon technology. Advancement of MEMS materials and manufacturing processes have enabled the fabrication of piezoelectric transducers with favourable features such as enhanced electromechanical coupling factor, piezoelectric coefficient, flexibility, stretch-ability and integrate-ability for diverse applications. Different MEMS configurations for PEH have been developed like cantilever, beam, zigzag beam, patches, diaphragm, cymbal, s-shaped or ring-shaped transducers. It is anticipated that many electronic devices are powered by piezoelectric energy harvesters. Piezoelectric materials can be made into thin film form, nanoparticles, nanowires or in layer stacks and may be categorised as inorganic, organic and composite/multilayer. 

#### 2.2.1. Inorganic Piezoelectric

**Figure 3:**
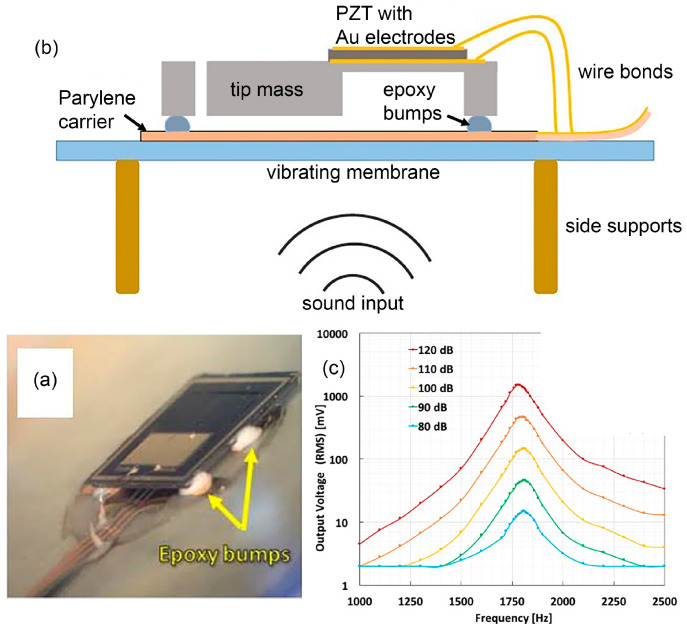
(**a**) Side view of a MEMS acoustic energy harvester chip attached on a carrier with epoxy bumps. (**b**) The schematic view of the piezoelectric transducer on the ear drum mimicking membrane. (**c**) Measured frequency sweep results of the prototype chip. Modified from [46].

Lead zirconate titanate (PZT) is the most well-known commercialised inorganic polycrystalline ceramic piezoelectric material that possesses perovskite characteristics. In [46], a MEMS acoustic energy harvester has been developed to utilise sound as the mechanical energy source in a fully implanted cochlear implant system (Figure 3a). This in vivo piezoelectric energy harvester is positioned on the tympanic membrane (eardrum) and transduces the incoming acoustic waves into electrical signals that can power up the hearing system. In Figure 3b, a PZT thin film layer (sandwiched in between gold electrodes) is deposited on a silicon (Si) cantilever and the whole structure is placed on a vibrating membrane made of ~40 μm thickness parylene film that mimics the ear drum. The top and bottom electrodes collect the generated electric charge from PZT, due to direct piezoelectric effect, and transfer it to the external load. The cantilever has a tip mass at the free end with a resonance peak of 1780 Hz. The PZT layer generates root mean square (RMS) voltage of 1.51 V with 150 Hz bandwidth at 120 dB acoustic input, which is sufficient to power up the signal processing circuits of CIs (Figure 3c). Maximum rectified output power was 16.25 μW with an open circuit direct current voltage of 2.47 V. The attained power density of 1.5 mW/cm^3^ was the highest amongst other MEMS acoustic energy harvesters. The developed chip can also be used to stimulate the auditory nerves, demonstrating a dual function of energy harvesting and acoustic sensing for a hearing system.

**Figure 4:**
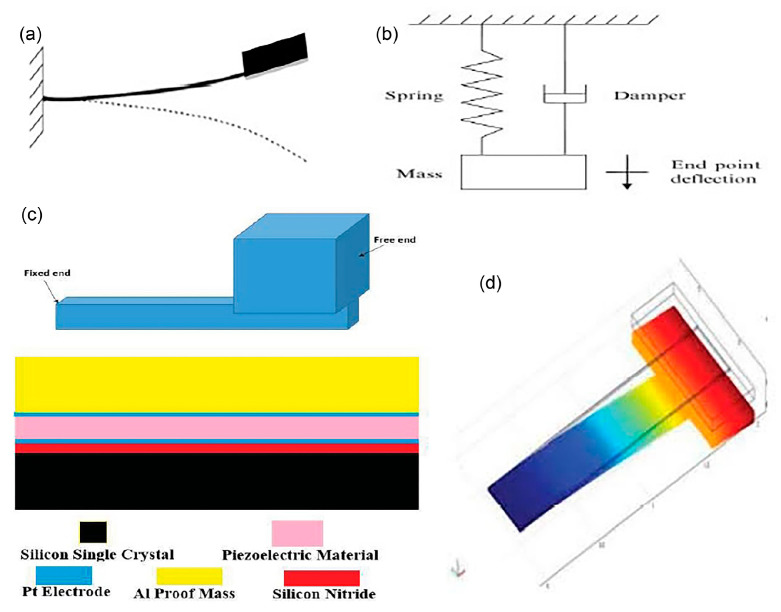
(**a**) Cantilever beam with top proof mass and (**b**) the equivalent spring-mass-damper system. © 2013 IEEE. Reprinted, with permission, from [47]. (**c**) The cross section of a T-shape cantilever beam with top proof mass and (**d**) its first modal shape. © 2019 IEEE. Reprinted, with permission, from [48].

In [47], a piezoelectric micro-power harvester has been designed for artificial cochlea. The energy harvester consists of a rectangular silicon (Si)/silicon nitride (Si_3_N_4_) cantilever and SU-8 photoresist top proof mass as a spring-mass-damper system with a single degree of freedom (Figure 4a,b). The active piezoelectric layer of platinum/PZT/platinum is proposed for d_31_ (piezoelectric coefficient) mode of operation where the bottom platinum (Pt) electrode prevents lead from PZT to migrate to silicon/silicon nitride during poling process. Finite element analysis estimates the resonant frequency for six modes of operation and its respective modal shape. Maximum harvesting output of 23 nW is simulated at the first mode, 589 Hz with a total cantilever displacement of 6 µm. The maximum output power occurs at the first mode while other modes demonstrate non-uniform cantilever bending. 

For maximum output power generation, the cantilever must resonate at its designed natural frequency, by which the surrounding sound input, like blues music, can resonate the cantilever with the natural frequency of 589 Hz. Additionally, higher power generation can be achieved by making the cantilever have a higher natural frequency, higher quality factor and larger mass of the top proof mass. Subsequently in [48], a T-shape cantilever beam has been designed with aluminium (Al) top proof mass (Figure 4c) that operates at 1334.09 Hz on its first modal frequency (Figure 4d). 

**Figure 5:**
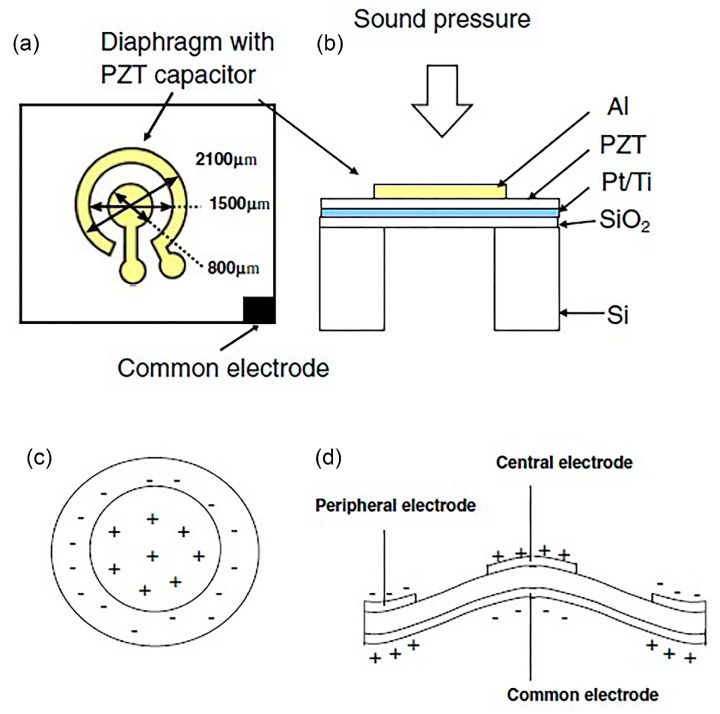
(**a**) Top view and (**b**) cross sectional view of the PZT diaphragm energy harvester. (**c**) Possible charge distribution at first mode and (**d**) the conceptual schematic of peripheral and central energy harvesting [49]. Copyright (2011) The Japan Society of Applied Physics.

An acoustic piezoelectric energy harvester of diaphragm configuration with a cavity underneath is fabricated to scavenge power from sound input [49]. The thin film PZT MEMS acoustic energy harvester possesses dual top electrodes that manipulate different polarisation charges on the surface of the vibrating PZT diaphragm, at the first mode. In Figure 5a,b, the diaphragm is made of Al/PZT/Pt/titanium (Ti)/silicon dioxide (SiO_2_). The top Al electrode covers independently the peripheral surface and central surface of the PZT diaphragm (Figure 5c,d). Power of 52.8 pW and 42.5 pW are generated at the peripheral and central, respectively at 4.92 kHz and sound pressure level (SPL) of 100 dB. A maximum power density of 23.8 µW/m^2^ has been reported at 100 dB SPL.

**Figure 6:**
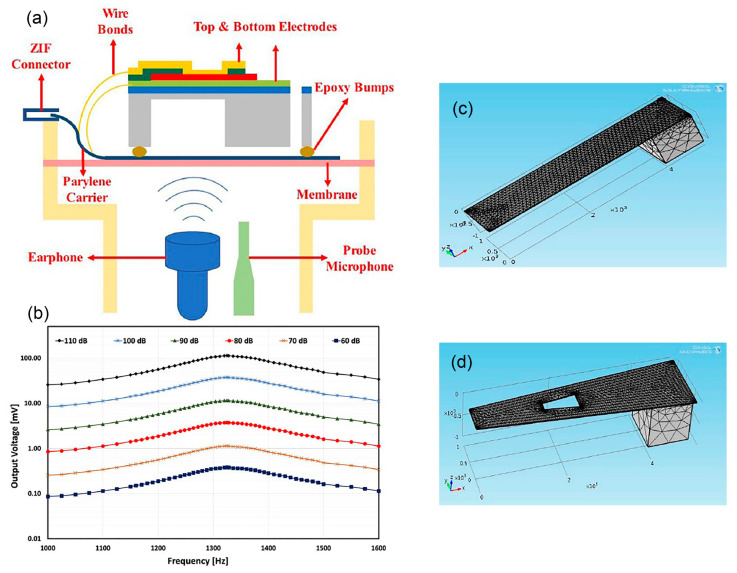
(**a**) The acoustic characterisation setup and (**b**) the measured acoustic results of a PZT energy harvester at sound pressure level from 60 dB to 100 dB SPL. Reprinted from [50], Copyright (2018), with permission from Elsevier. The meshed (**c**) rectangular and (**d**) tapered perforated ZnO cantilever structure with proof mass. The tapered perforated structure has the same area as the rectangular cantilever and the narrower side is fixed while the broader side is free to vibrate. © 2017 IEEE. Reprinted, with permission, from [51].

In [50], another PZT cantilever thin film has been fabricated and attached onto the acoustically vibrating membrane resembling the behaviour of an eardrum (Figure 6a,b). The energy harvester generates 114 mV output at 110 dB SPL and 1325 Hz. The transducer has high sensitivity level of 391.9 mV/Pa at 900 Hz, which enables the generation of strong and easily processed readout circuit signals.

In [51], zinc oxide (ZnO) is chosen as the piezoelectric material for the MEMS piezoelectric energy harvester in cochlear implant due to its biocompatibility characteristics. The ZnO/Si cantilever is placed on the eardrum with a preferable length of not more than 5 mm. The attachment of proof mass reduces the natural frequency of the cantilever to audible frequency range. The fundamental frequency of adult male voice speech is typically around 125 Hz. Thus, the energy harvester is designed to work around this frequency region so that it can respond readily with the surrounding voice sound. A finite element analysis is performed to compare the performance between a rectangular cantilever and a tapered cantilever with perforation (Figure 6c,d). The tapered and perforated design helps to further reduce the cantilever’s natural frequency without increasing the cantilever length, besides improving the average strain value and increasing the voltage output. The peak output voltage of the perforated tapered cantilever structure is 0.75 V at the resonant frequency of 126 Hz while the rectangular cantilever of the same area generates smaller output voltage of 0.35 V at higher resonant frequency of 139.4 Hz. 

Inorganic piezoelectric ceramics like PZT and barium titanate (BaTiO_3_) have a lack of integration feasibility with the conventional CMOS technology processing due to high electric field poling process and high temperature annealing treatment needed for the thin film piezoelectric layers. PZT possesses superior piezoelectricity performance, however poling is needed for this ferroelectric ceramic to achieve excellent piezoelectric property whereby a very strong electric field, typically in the order of 50 kV/mm has to be applied on the material to align its domains [52,53]. Other material structures which were integrated together on the same substrate will also be subjected to poling, causing material breakdown. ZnO and aluminium nitride (AlN) thin film layers possess low dielectric permittivity and high piezoelectric voltage coefficient with wurtzite crystal structure. Although perovskite PZT crystals layer has better piezoelectric properties than the wurzite’s, ZnO and AlN are easily manufactured into energy harvesters without the implementation of poling process to attain piezoelectricity. The wurzites are non-ferroelectric inorganic piezoelectric materials by which the polar axis is determined by the crystal orientation. Both ZnO and AlN can be grown to have a permanent polarisation effect along its c-axis crystallographic direction, achieving high piezoelectricity [54]. Therefore, the thin film layers can feasibly be deposited and processed on IC circuits without having to undergo the high electric field poling process. 

Ceramic like PZT is usually sintered or annealed at high temperature 600–700 °C, 4 to 6 h to densify the thin film layer and form the desired perovskite crystalline structure. Temperature can damage parts of the device including metal layers and polymers. This will hinder the integration of ceramic-based transducers with low temperature and soft materials. Radio frequency (RF) magnetron sputtering technique can be used to grow nanolayer of thin film piezoelectric oxides or nitrides at low temperature [55]. During the sputtering process, the device temperature will generally increase a little (~60 °C) due to plasma heating [56]. Therefore, ZnO and AlN can be grown at low temperature and are suitable to be integrated with flexible polymeric substrate and other low temperature CMOS processes. The desired highly c-axis AlN crystals have been reported to be grown on molybdenum electrodes [55,57]. In addition, there is another issue related to the implementation of PZT for in vivo PEH. PZT contains lead which is harmful to the human body and the environment. ZnO, AlN and BaTiO_3_ are non-toxic and human friendly materials, unlike PZT [51]. High level of material toxicity in medical implants can cause kidney damage and fatality, leading to hazardous impacts after being implanted. Thus, the choice is between designing a biocompatible material for the energy harvester or designing a hermetic seal to encapsulate PZT [25].

#### 2.2.2. Organic Piezoelectric

Piezoelectric polymers like polyvinylidene fluoride (PVDF) and its co-polymers like polyvinylidene fluoride-trifluoroethylene (P(VDF-TrFE)) are organic piezoelectric material that are naturally durable, lightweight, flexible, easily processed, biocompatible, possess low permittivity and have low acoustic impedance. The piezoelectric material can be implanted in the scala tympani of the cochlea and uses the cochlea’s natural mechanofluidic movement to generate electrical power. The in vivo implanted piezoelectric energy harvester is immersed in the cochlear perilymph fluid, in the path of the pressured waves. The piezoelectric polymer in between two electrodes generates voltage that can directly stimulate the nerves (replicating hair cells) or power up other parts of the CI like microphones or signal conditioning circuits. Basically, the self-powered device can replace the stimulation electrode in CI with the source of energy originated from the inside of the cochlea itself, initiated by the incident sound pressure waves. A proper mechanical functioning of the outer, middle and inner ear is necessary for the energy harvester to work.

**Figure 7:**
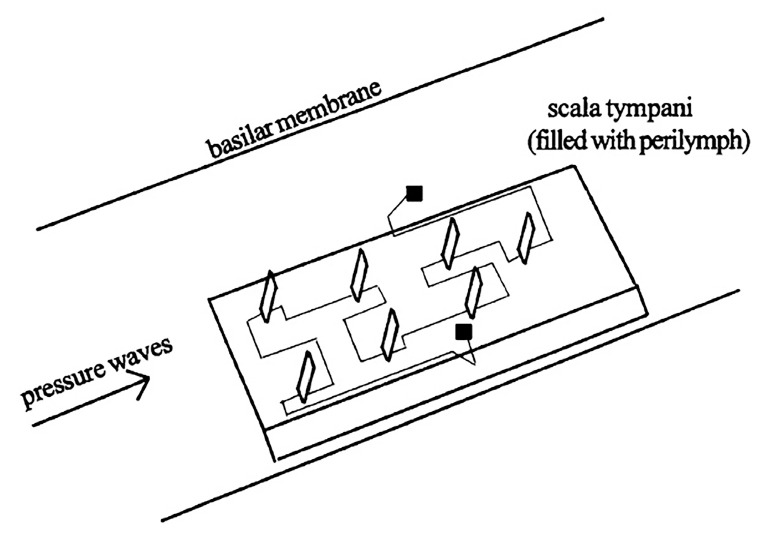
The schematic of vertical PVDF cantilevers device positioned in the scala tympani of a cochlea [58]. Reprinted by permission from Springer Nature Customer Service Centre GmbH: Springer Nature MRS ONLINE PROCEEDINGS LIBRARY Considerations in the Development of a Piezoelectric Transducer Cochlear Implant, N. Mukherjee et al., COPYRIGHT (2011).

High specific acoustic impedance mismatch between perilymph fluid and piezoelectric ceramic causes smaller voltage charges to be generated. In [58], vertical cantilevers made of PVDF are proposed to be embedded in a flexible insulating substrate, fitted into the cochlea’s scala tympani (Figure 7). PVDF’s acoustic impedance is close to that of saline, is mechanically flexible and produces better voltage sensitivity compared to ceramics. Using its flexural/bending mode, the vertical piezoelectric cantilevers are bent by the incoming pressured waves, eliciting charges on the electrodes. A voltage response of more than 1 mV is estimated from all cantilevers operating under the average intra-cochlear pressure conditions. 

Polyvinylidene fluoride (PVF_2_) offers the highest sensitivity of piezoelectric bending amongst other organic piezoelectric polymers [59]. Mukherjee et al. have developed PVF_2_ cantilevers with nickel (Ni) and copper (Cu) as the electrodes [59]. PVF_2_ is stretched 300% to attain its piezoelectric β phase characteristics. Underwater acoustic measurement reveals three bending modes under 1 kHz that the cantilevers exhibit, which are strongly correlated with the cantilever length and thickness. The sensitivity measurements of Ni/PVF_2_/Cu cantilevers demonstrate the amount of voltage generated from the cantilevers with respect to sound pressure in fluid environment. The voltage generation from this piezoelectric device depends on the applied sound pressure level where the sensitivity is measured to be 0.15 mV/Pa at 125 dB SPL and 0.05 mV/Pa at 150 dB SPL.There is a triple decrease in sensitivity with respect to the ten times increase in the applied acoustic pressure waves. Afterwards, a conducting polymer polypyrrole (PPY) is used as the electrodes for the similar-sized PVF_2_ cantilevers in order to gain more flexibility in the cantilevers and to eliminate the acoustic impedance mismatch between water and cantilevers due to the usage of metal as electrodes. Similar high sensitivity levels are generated such as the ones measured from the metal electrodes, and at some frequencies, better sensitivities are achieved. PPY is a promising electrode material for in vivo fluidic acoustic piezoelectric energy harvesters implanted in a cochlea. 

**Figure 8:**
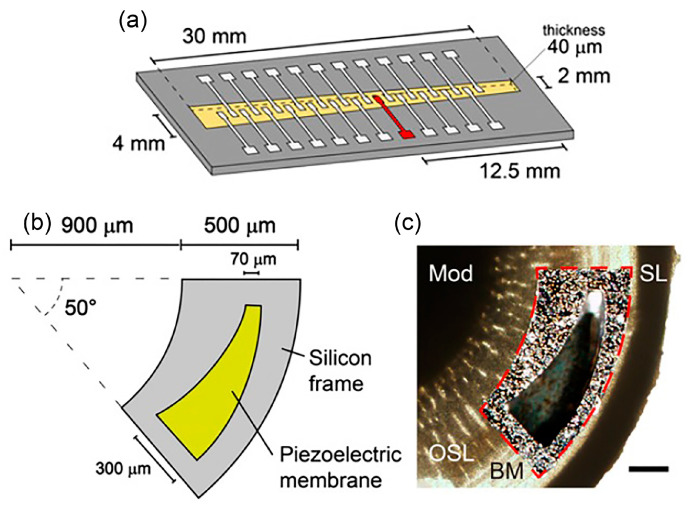
(**a**) The schematic diagram of PVDF membrane (yellow) with 24 aluminium top electrodes fabricated on a trapezoidal slit of a stainless plate. (**b**) The schematic drawing of the implantable P(VDF-TrFE) membrane and (**c**) the integrated optical image of the implanted P(VDF-TrFE) membrane in the basal turn of the cochlea [60].

Inaoka et al. adopted PVDF and P(VDF-TrFE) membranes to mimic the function of auditory hair cells that sense BM vibration and transduce it into electricity [60]. In Figure 8a, a PVDF membrane of 40 µm thickness with aluminium (Al) electrodes has been fabricated on a slit of a stainless plate. The amplitude of piezoelectric output is measured to be 16 µV at 90 dB SPL in silicone oil. The maximum electrical output is generated at the maximum vibration location on the membrane. The fabricated Al/PVDF/Al membrane is able to harvest sound energy and produces electrical signals in fluid condition but the device is too large in size to be fitted in a cochlea. A life-sized 3 µm thickness P(VDF-TrFE) membrane is then fabricated on a 300 µm silicon frame with slit, before being implanted into the scala tympani in the basal turn of an intact guinea pig cochlea (Figure 8b,c). Ex vivo measurement shows the membrane’s largest vibration amplitude of 642 nm at 3 kHz, 101.7 dB SPL. The platinum/P(VDF-TrFE)/gold membrane generates 0.14 mV–5.88 mV at 100 dB SPL. 

Human organs and tissues are physically non-planar and soft, some are stretchable like muscles and skin, and they possess low acoustic impedance and low frequency of the biomechanical motion. The in vivo piezoelectric energy harvester in implants should have some degree of flexibility in order to synergically couple with the continuous biomechanical movement and for proper attachment/mounting onto the organ structure. The organic piezoelectric material can be made into large area of thin film layer and can conform well onto the surface of curved structures due to its large elastic compliance. For inserting a piezoelectric membrane into the curving cochlear duct for example, the flexible organic piezoelectric layer can accommodate the curvy inner ear anatomy. Although the organic piezoelectric material can work at a lower frequency compared to ceramics, there is still a concern regarding the very low natural frequencies of the biological motions and audible sound. The human heart beats around 39 Hz while an adult male voice makes the tympanic membrane or basilar membrane to vibrate around 125 Hz. The energy harvester often operates at higher resonance frequency. Adding a proof mass and increasing the transducer’s size are usually done to reduce the resonance frequency. However, the device still needs to be small to avoid causing discomfort or interference with the organ’s function. 

The organic piezoelectric polymer exhibits inferior piezoelectricity performance compared to the ceramics. PVDF is a ferroelectric material that requires poling in order to attain β molecular dipole alignment for the material to exhibit piezoelectric behaviour. PVDF can be made into micro/nano-fibres using electrospinning technique [52]. PVDF in its electrospun fibres form is five times more sensitive to acoustic signals than that of PVDF in thin film solid form with the reported enhanced dielectric and piezoelectric constant by an order of two [45,53]. In the electrospinning process, the PVDF layer is stretched mechanically and subsequently a large static electric field is applied at elevated temperatures. Stretching aligns amorphous strands in PVDF in planar direction, allowing easy rotation of crystallites by an electric field afterwards. Therefore, simultaneous fibres formation and poling process occurred during electrospinning. In situ poling during fibre formation produces fibres with high percentage of β and thus poling step is unneeded after electrospinning. The desired β phase in PVDF is formed during electrospinning due to the applied high voltage that aligns the existing molecular dipoles in PVDF [61,62]. 

The low power density of piezoelectric polymers can be overcome by introducing porosity in the material structure that reduces the effective dielectric permittivity and increases the piezoelectric voltage constant. In [63], nanopores are formed in the electrospun P(VDF-TrFE) nanofibres and the fabricated P(VDF-TrFE) energy harvester yielded as high as 500-fold greater output power at 45% porosity compared to the nonporous ones. Another technique that can be employed to enhance the piezoelectric conversion efficiency of vibration energy in piezoelectric polymers is introducing the phononic crystals [64,65]. Local cavity can be created by removing a crystal from a perfect sonic crystal. A PVDF piezoelectric film was then placed in the cavity of a sonic crystal to convert the acoustic energy into electrical energy at the resonance frequency of the cavity. The generated voltage in the cavity has been measured to be 600 times greater than the ones without cavity. Overall, the development of organic piezoelectric polymers for hearing system’s energy harvester has become a subject of few recent studies even though insufficient voltage generation has been reached so far using organic piezoelectric materials alone. 

#### 2.2.3. Piezoelectric/Polymeric Nanocomposite and Multilayer

It is known that the organic piezoelectric materials possess inferior piezoelectric properties compared to the inorganic piezoelectric. The development of hybrid piezoelectric/polymeric nanocomposite or the structuring of piezoelectric/polymeric multilayer can yield better piezoelectric behaviour compared to the organic piezoelectric materials alone [62]. For example, nano-sized ceramics (PZT) can be dispersed in a piezoelectric polymeric matrix (PVDF) and the composite is found to exhibit similar flexibility characteristics as the piezoelectric polymer but with a higher piezoelectric coefficient [29,53]. The nano-sized reinforcement or nanofillers which can be conductive, non-conductive, piezoelectric or ferromagnetic serve as the nucleation and crystallisation sites. Also in [66], Bhavanasi et al. reported on the enhancement of piezoelectric energy harvesting performance in the thin film bilayer structure made of poled P(VDF-TrFE) and graphene oxide (GO) compared to the poled monolayer P(VDF-TrFE). There are multiple factors that might be affecting this phenomenon including the electrostatic and piezoelectric contribution from GO, residual tensile stress, enhanced Young’s modulus of the bilayer film and the presence of space charge at the interface of P(VDF-TrFE) film and GO film [66,67]. Interfacing high Young’s modulus and dielectric constant of GO with ferroelectric materials leads to efficient transfer of mechanical and electrical energy, and thus improves the energy harvesting performance.

**Figure 9:**
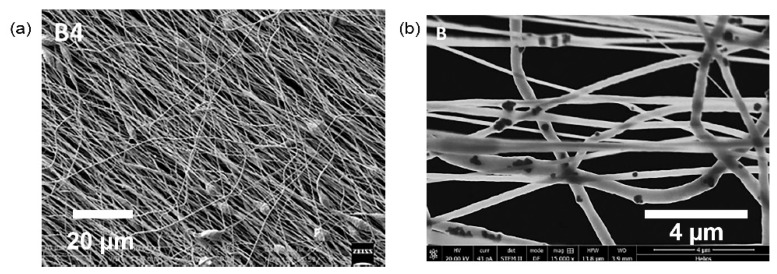
(**a**) The morphology of electrospun BTNP/PVDF fibres at 20/80 weight composition, collected on a rotating disk at high tangential velocity of 22 ms^−1^. (**b**) Dispersion of BTNPs inside the electrospun PVDF fibres where beads are induced by the presence of BTNP aggregation [68] Copyright (2017), with permission from Elsevier.

In [68], a piezoelectric ceramic/polymer nanocomposite based on ultrafine PVDF fibres and BaTiO_3_ nanoparticles (BTNPs) is developed to mimic hair cell function. Thin meshes of BTNP/PVDF fibres are created using electrospinning technique, attaining an optimised 20/80 weight composition ratio. During electrospinning, the increase of collector velocity to 3000 revolutions per minute (rpm) improves fibre alignment and an enhanced PVDF piezoelectric β phase is obtained. Figure 9 shows the morphology of the aligned ultrafine fibres for BTNP fillers in PVDF matrices. The greater weight composition of BTNP results in higher piezoelectric coefficient of the nanocomposite. BTNP/PVDF fibres generate piezoelectric coefficient of 0.5 mV per Newton at 0/100 weight composition to 1 mV per Newton at 20/80 weight composition. The BTNP/PVDF fibres are biocompatible towards cochlear epithelial cells and this piezoelectric nanocomposite can be used to harvest energy for CI. The neural cells adhere well to the composite fibres, demonstrating favoured neural cell contact. 

**Figure 10:**
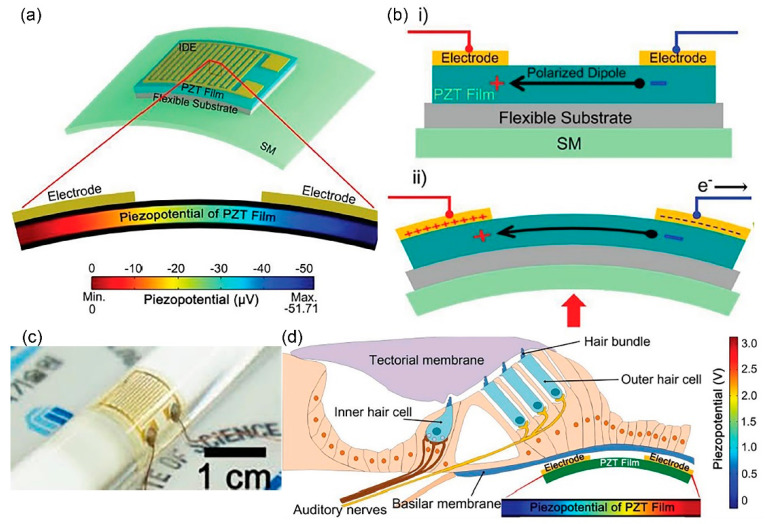
(**a**) Gold IDEs patterned on the flexible PZT film and the simulated potential distribution in PZT film between the adjacent electrodes. (**b**) The principle operation of piezoelectricity generation in iPANS device (i) before and (ii) after bending deformation. (**c**) The attachment of an iPANS device on a 1 cm curvature radius of glass rod. (**d**) The conceptual schematic of flexible iPANS device insertion under BM in a mammalian cochlea. The upward bending at 600 nm due to sound wave vibration generates 3 V of piezoelectric potential [69].

In [69], a flexible inorganic piezoelectric acoustic nanosensor (iPANS) is fabricated using uniform PZT thin film bonded on a flexible thermoplastic polymer substrate, polyethylene terephathalate (PET) using ultraviolet cured-polyurethane (PU) as an adhesion layer. In Figure 10a, a gold (Au) metal layer is deposited on the piezoelectric PZT layer and patterned into interdigitated electrodes (IDEs). The whole structure of flexible iPANS generator is then firmly fixed onto artificial silicone-based basilar membrane (SM) using double sided adhesive tapes. The simulation in Figure 10a demonstrates a generation of 51.71 µV piezoelectric potential in response to 15 nm vertical displacement of SM. Basically, the bending deformation of device leads to change of the dipole state inside PZT, causing piezoelectric potential difference in PZT and thus converts sound-driven vibration into electricity. Figure 10b illustrates (i) the existing polarised dipole from the poling process which is aligned parallel to the surface of PZT between the adjacent electrodes and (ii) when the device structure is bent by sound driven pressure, there is a change of dipole moment. A piezoelectric potential difference between adjacent electrodes is generated instantaneously and the electrons flow to the external circuit. The flexibility of iPANS is demonstrated in Figure 10c in that the device can be wrapped around the glass rod of 1 cm curvature radius. A flexible iPANS has been measured to transduce sound vibration at SM vertical displacement of ~7.6 nm into electrical signals of ~59.7 µV at 40 dB SPL, 1 kHz. Theoretically, the placement of iPANS beneath a basilar membrane (Figure 10d) could mimic the artificial hair cells. The basilar membrane responds to sound stimulation and vibrates vertically at 600 nm generating 3 V of piezoelectric potential. 

**Figure 11:**
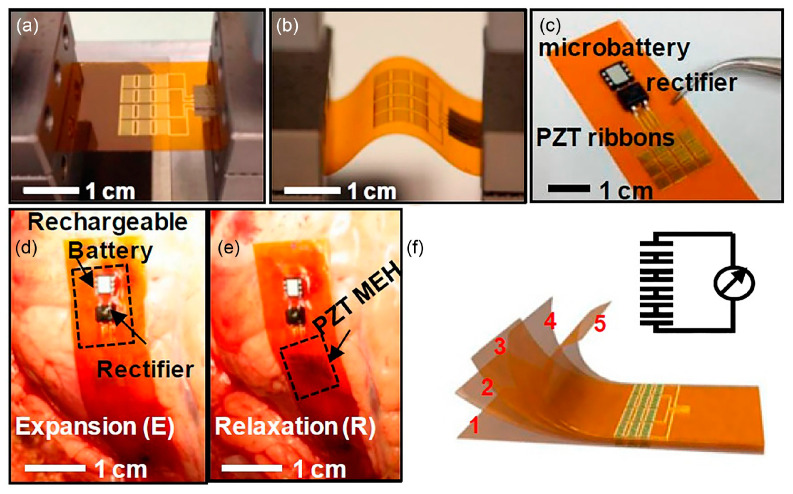
Twelve groups of 10 MEHs clamped on a bending stage in (**a**) flat and (**b**) bent config-uration. (**c**) The PZT/PI MEHs device is connected and integrated with a rectifier and rechargea-ble microbattery. (**d**,**e**) The integrated MEHs is attached onto the right ventricle of bovine heart during expansion and relaxation. (**f**) The illustration of a multilayer stack of five PZT/PI MEHs sheets connected in series and the equivalent schematic circuit [70].

Dagdeviren et al. have developed a mechanical energy harvester (MEH) using a combination of PZT and polyimide (PI) layer [70]. The PZT is in ribbons formation, created in between the top gold electrode and bottom platinum electrode. Polyimide serves as the flexible substrate and biocompatible encapsulation layer. 10 MEHs are fabricated on a polyimide sheet and electrically connected in parallel. In Figure 11a, twelve groups of 10 MEHs are connected in series on a polyimide sheet to increase the output voltage. Figure 11b demonstrates the deformation of a MEHs sheet which will induce a distribution of strain in PZT ribbons. The device can be integrated monolithically with a rectifier and battery for power generation and storage (Figure 11c). The in vivo device performance is evaluated by measuring its output voltage on bovine and ovine heart of varying heart rates, also on lung and diaphragm during respiration. Figure 11d shows the attachment of the energy harvester with a rectifier and rechargeable battery onto a bovine heart. From the organ’s biomechanical motions, the open circuit output voltages were measured in the range of 2 V to 4 V. The PZT/PI MEHs sheet is capable of generating voltage using the natural contractile and relaxation motion of the heart, lung and diaphragm, indicating the readiness of the flexible device in harnessing energy from different locations across the body. Heartbeats and lung/diaphragm respiration are inexhaustible sources of energy during a patient lifespan. Stacking multiple MEHs sheets (Figure 11e) has been found to increase the output power. The time-averaged power density of using five layers of PZT/PI MEHs sheet can reach up to 1.2 µW/cm^2^ which is adequate for operating a cardiac pacemaker, with or without battery assist. This device is a promising energy harvester for hearing devices.

**Figure 12:**
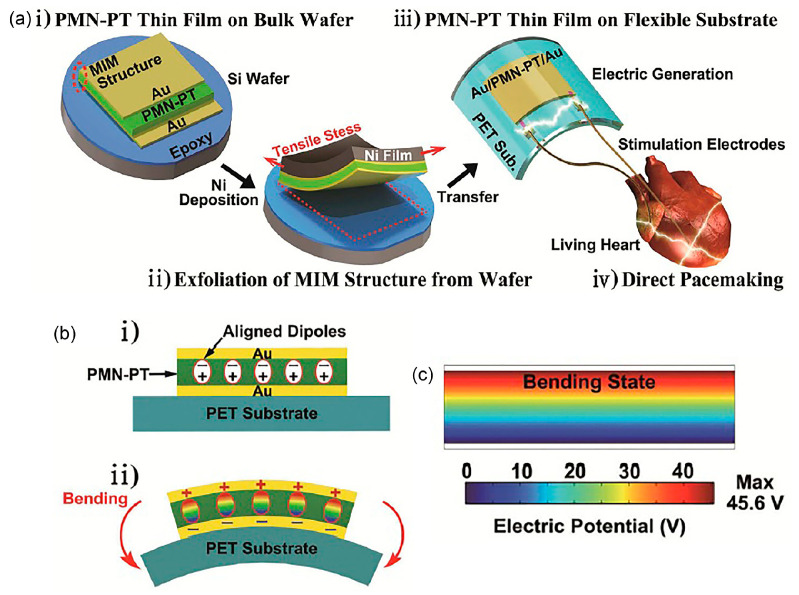
(**a**) Schematic illustration of the fabrication process and biomedical application of the flexible PMN-PT/PET piezoelectric energy harvester. (**b**)The piezoelectrical generation of the flexible PMN-PT/PET thin film (i) before and (ii) after bending. (**c**) The simulated piezoelectric potential distribution in PMN-PT thin film under tensile strain of 0.36% [71].

In [71], an efficient flexible piezoelectric energy harvester is fabricated using thin film single crystal lead magnesium niobate-lead titanate (PMN-PT) on a flexible thermoplastic polymer substrate, PET. The new generation single crystalline PMN-PT has been long known to exhibit exceptional piezoelectric charge constant of four times higher than PZT. High electric field is applied to pole PMN-PT before depositing onto the gold electrode layer. In Figure 12a, a gold/PMN-PT/gold thin film (MIM structure) on a bulk substrate is transferred to the flexible substrate without mechanical damages via the stress-controlled exfoliating process. A tensile nickel (Ni) layer is deposited on device and the whole MIM structural layer will be peeled off from the substrate spontaneously and uniformly. The device layer is then transferred onto the PET substrate. The freestanding flexible gold/PMN-PT/gold thin film on PET demonstrates conformal contact on curved subcutaneous layer and corrugated organs. A self-powered pacemaker is achievable from this device. In Figure 12b, the perpendicularly aligned dipoles in PMN-PT due to poling will induce charges on the electrodes when deformed. Piezoelectric potential is generated and electrons flow to the external load. Under periodic motion of bending and unbending, positive and negative electrical signals are generated from device. Figure 12c shows the estimated 45.6 V of piezoelectric potential using finite element analysis. PMN-PT layer converted tiny biomechanical motion into electric energy with current signal of up to 145 μA and output voltage of 8.2 V over a working area of 1.7 cm × 1.7 cm. The experimentally measured 8.2 V from the PMN-PT energy harvester was lower than the simulated result of 45.6 V presumably due to voltage drop from internal leakage paths and/or charge loss in the structure.

**Figure 13:**
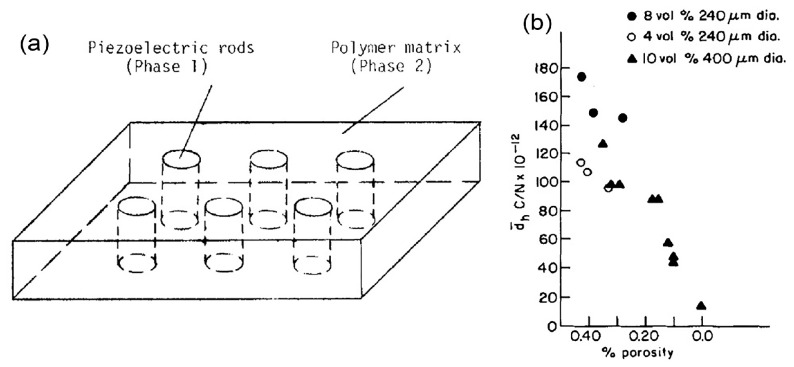
(**a**) Composite with 3-1 connectivity in which rods of piezoelectric are embedded in a three-dimensionally continuous polymer. PZT rods can be poled along the length and thus the direction of the PZT/epoxy composite is along the direction of the rods alignment [73]. (**b**) The measured hydrostatic piezoelectric constant of a composite PZT/foamed PU as a function of porosity in PU matrix [74].

The development of in vivo energy harvester implanted in a cochlear duct poses a challenge of low generated voltage from a piezoelectric PZT or BaTiO_3_ thin film due to fluidic surrounding. The effective piezoelectric constants become notoriously small under hydrostatic condition. Due to high acoustic impedance mismatch between ceramics and perilymph fluid, smaller acoustic pressure is sensed by the device as the pressure transmitted towards the device is a small percentage of the actual pressure in air. A polymer coating on piezoelectric layer as an impedance matching layer can partially offset the high impedance mismatch. Piezoelectricity increases monotonically with respect to the increase of polymer coating thickness and the substantial increase in sensitivity at audio frequency operation is only obtained at large polymer layer thickness [58,72]. In [73], Klicker et al. have fabricated 3-1 nanocomposites of extruded PZT rods embedded in a compliant epoxy polymeric matrix (Figure 13a). It is reported that the hydrostatic piezoelectric coefficient is enhanced in the composite layer compared to plain PZT. Generally, higher hydrostatic piezoelectric constant is achieved at higher volume fraction of PZT, smaller rod diameter of PZT and higher PZT/epoxy composite thickness. Composite with 10% of PZT offers two times larger of hydrostatic piezoelectric coefficient and 25 times larger of hydrostatic voltage coefficient compared to bulk PZT. Further improvement in hydrostatic coefficients has been accomplished using a PZT/foamed PU composite [74]. It is a piezoelectric nanocomposite with 3-1 connectivity and a foamed PU matrix. The effect of porosity in PU on the hydrostatic piezoelectric constant of the composite is measured in Figure 13b. In general, the hydrostatic piezoelectric constant increases rapidly with volume porosity. Hydrostatic piezoelectric sensitivity of the rod ceramics/polymer matrix composite with >30% porosity increases more than 10 times greater than a similar composite with no porosity matrices.

Bulk inorganic piezoelectric ceramics are physically brittle and rigid. Although the material possesses superior piezoelectric performance, it is difficult to secure flexibility and conformability owing to its brittle and rigid characteristics. The human organs evolve with respect to age and the surfaces are usually corrugated and curved. The in vivo inorganic PEH might be in incongruent contact with the non-planar structure of organs due to the rigidity and brittleness of ceramics. For cochlear devices, it is crucial for the energy harvester to be firmly attached onto the tympanic membrane or basilar membrane. Contrariwise, the highly flexible organic piezoelectric exhibits poorer piezoelectricity characteristics. Therefore, the nanocomposite or multilayer of inorganic piezoelectric/organic piezoelectric may attain both mechanical flexibility and excellent piezoelectric properties. The elastomeric behaviour of the organic piezoelectric polymer can protect fragile ceramic by increasing its effective strength and improves the robustness. In addition, the polymeric material layer is capable of protecting the inorganic piezoelectric from environment like humidity and encapsulating the piezoelectric material’s toxicity from reaching the human body. Table 3 presents the detailed and comprehensive comparison of different types and forms of piezoelectric materials used for in vivo implantable energy harvester with potential application in powering cochlear devices.

### 2.3. Other Consideration Aspects of In Vivo PEH for Totally Implanted Cochlear Device

For cochlear devices, the batteries can be absolutely eliminated in order to adopt in vivo energy harvester, creating a self-powering system. In a fully implantable cochlear device, the energy harvester that gains energies from the internal organs needs to deliver power to the microphone, amplification electronics, signal conditioning circuits, energy storage and stimulation electrode array for CI or FMT for MEI. 

An energy storage device can be connected electrically to the in vivo PEH as an energy recovery element that extracts and stores the non-used energy from piezoelectric materials for the subsequent usage or in other CI stages. In vivo PEH produces power of alternating current (AC) behaviour with high voltages, low currents and very large internal impedance. In terms of power management and storage aspects, these characteristics complicate the storage efficiency of the generated power. Attention should be directed towards minimising the impedance mismatch between the energy harvester and storage components and designing the rectification circuitries that can optimise the energy utilisation efficiency in order to avoid large power lost [52]. In biomedical applications, similar material properties to the energy harvesters, such as flexibility and small size are desired for the storage components.

All of these separate components (except for the electrode array and FMT) can be fabricated into one chip using CMOS processes. The key point is that the materials used for microphones, amplification electronics, signal conditioning circuits, energy harvesters and energy storage are all compatible with the process. Piezoelectric/polymeric-based microphone and energy harvester can easily be integrated with the other components and the integration drastically reduces the total volume of a hearing system. 

## 3. Implantable Microphones for Totally Implanted Hearing Device System

A microphone in a hearing device captures acoustic audible sound energy and transforms it into electricity. Just like the energy harvesters, there are different types of mechanoelectric transduction mechanisms that can be adopted for the microphones. The electrostatic, optical, piezoelectric and electromagnetic-based sensors have been studied towards the development of totally implanted hearing system which can be implanted subcutaneously, in the middle ear or in the inner ear. Technically, different types of sensor can be utilised for sound sensing, either microphones, accelerometers, displacement sensors or force sensors [22]. The microphone’s configuration is usually in the form of a membrane diaphragm. Subcutaneous microphone can cover large surface area and thus high sensitivity but it easily picks up body noise and suffers from sound attenuation due to sound filtering effect from the skin. Implantation inside the ear canal or on the ossicular chain in the middle ear do not have to face these issues but may experience feedback problems between sound source and microphone. 

### 3.1. Mechanical Energy Sensor

Among the mentioned mechanoelectric transduction methods, the systems based on electrostatic and piezoelectric are preferred for totally implantable microphones due to the easier integration with the electronic circuits, although these electrical systems are more susceptible to noise whereas the optical-based microphones generate clear signals with no noise [75]. The piezoelectric acoustic sensor gives smaller power output of a few µW to 100 µW compared to the electrostatic-based microphone. However, high bias voltage is needed for the electrostatic-based microphone while the piezoelectric-based microphone has an advantage of directly converting acoustical energy into electrical energy without any voltage source. Similarly, the optical sensing and readout mechanism are advantageous in terms of sensitivity but require a power source to drive the system. Triboelectric transduction method is another mechanism that can also be utilised to obtain a self-powered device without an external power source and thus it may work sustainably inside a human body. A triboelectric device is based on the coupling between contact electrification and electrostatic transduction in order to achieve the mechanical–to-electrical energy conversion [76].

**Figure 14:**
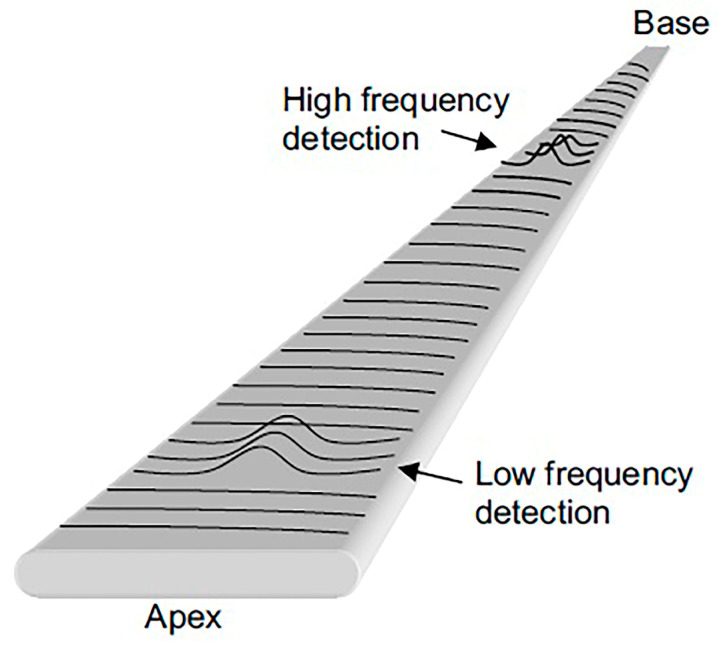
The schematic illustration of an uncoiled basilar membrane demonstrating the tonotopic organisation behaviour along the membrane length [77]. Reprinted by permission from Springer Nature Customer Service Centre GmbH: Springer Nature MICROSYSTEM TECHNOLOGIES MEMS design and modelling based on resonant gate transistor for cochlear biomimetical application, R. Latif et al., COPYRIGHT (2016).

The human cochlea possesses tonotopic arrangement response to frequencies as the BM is organised tonotopically. In the basilar membrane, the apex mainly responds at low frequency sound whereas the base is sensitive to high frequency sound signals, due to the varying rigidity along the membrane (Figure 14). The BM is in the shape of a trapezoid where the base close to oval window is thick with short breadth giving high rigidity whereas the apex is thin and long breadth giving high flexibility [77]. In the current commercialised cochlear devices, the sensed signals by microphones are filtered by the digital signal processor technology that divides speech into different frequency bands, attaining the tonotopic distribution of frequency. The flexible programmable strategies can adopt certain speech processing algorithms and coding that will extract and encode the right features from the sensed signals. 

Other than the membrane diaphragm, different forms of configuration and composition can be used for the microphone structure. It can be in the form of a cantilever array, beam array, membrane array or a trapezoid-shaped membrane which are designed geometrically to resonate at certain frequencies when stimulated with the mechanical acoustic waves and might be a single layer, bilayer or multilayer. The cantilever/beam/membrane resonator array can perform the passive mechanical frequency selectivity, mimicking the tonotopy of BM. A trapezoidal membrane with exponentially varying width is mainly inspired by the physical structure of the basilar membrane itself, which is trapezoidal in shape. BM has the characteristics of varying width, thickness and stiffness at different positions on the membrane. The cantilever length, beam thickness, rectangular membrane’s width, circular membrane’s diameter or trapezoidal membrane’s width are the parameters that can be varied to design the working frequency range. 

Bachman et al. describe on how an array of MEMS resonators like cantilevers and beams could copy not only the microphone but also partially the functions of a speech processor in CI [75]. The array is capable of splitting sound into its frequency sub-bands, bypassing the analogue-to-digital converter and subsequent digital processing. The array is basically performing the mechanical Fourier Transform at the front end of a CI. Each of the MEMS resonators in the array corresponds to different resonant frequency. The outputs from the array are individual electrical signal channel at a particular frequency point with its own individual amplifier. Each resonator possesses its own individual sophisticated frequency response profile. The device output can be applied directly to stimulate cochlea according to its tonotopic organisation characteristics. The array can be employed as the implantable microphone for CI and it is suggested to be positioned close to the eardrum.

The implantable sensor for a hearing device, either a microphone diaphragm, an accelerometer or the resonator array requires a broadband frequency response, but not extending to very low frequencies of less than 200 Hz, in order to minimise the response to vibrations produced by body movements [22]. The frequency range for a normal human communication is actually around 100 Hz–4 kHz. In noisy situations, capturing higher frequency sound in between 4 kHz and 8 kHz is essential for speech understanding. Ideally, the frequency range operation should therefore comprise of frequencies from around 100 Hz to 8 kHz with a flat frequency response within this range. However, it should be noted that the body sound interference between 100 Hz to 200 Hz limits the performance of an implantable sensor. The dynamic range of human hearing is from ~−5 dB SPL to 100 dB SPL between 3 kHz to 4 kHz whereas the dynamic range of human speech in English is ~60 dB. Most microphones are sensitive within the dynamic range of 30 dB SPL to 140 dB SPL, which corresponds to the sensor’s internal noise and the beginning of distortion/non-linearities. For middle ear implantation, the appropriate input dynamic range for the implanted microphone should be from 40 dB SPL to 100 dB SPL, applied at the tympanic membrane (20 µPa). Additionally, low power consumption of less than 1 mW and a miniature implantable microphone size not exceeding 2 × 2 × 2 mm^3^ is sought.

#### 3.1.1. Optical-Based Sensor

**Figure 15:**
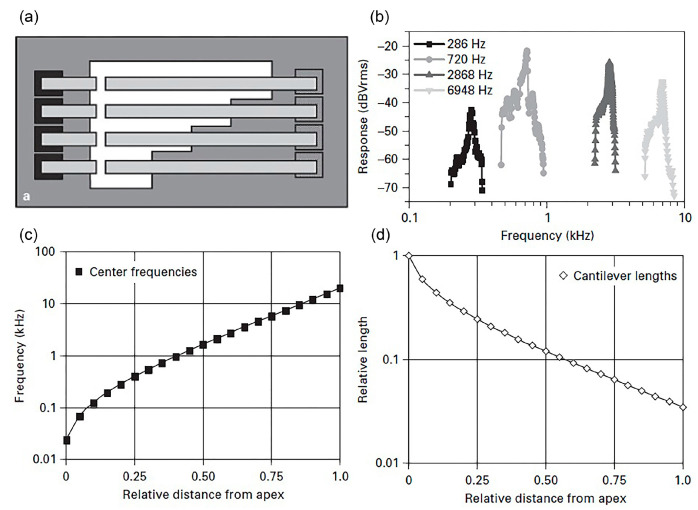
(**a**) 4-channel multiresonant microphone made of four free-standing cantilevers with different lengths. There is a 20 µm air gap between the cantilevers and the receiving light pipes. (**b**) The measured frequency response from the cantilevers at 70 dB SPL. (**c**) The tonotopic mapping characteristics of a human cochlea demonstrating the frequency point as a function of distance from apex of cochlea. (**d**) The corresponding length of a cantilever that can give the required resonant frequency $f_{c}$ at the frequency points [75]. Reprinted by permission from S. Karger AG, Basel.

Building an artificial cochlea that mimics the real cochlea’s functionality has led to the development of the front end transducer for cochlear devices. In [75], an optical-based microphone has been developed which consists of an array of suspended micromachined SU-8 epoxy polymeric cantilevers with length ranging from 2 mm to 7 mm, width of 100 µm and thickness of 40 µm. The cantilevers are sensed optically via photodetectors located at the exit end of the receiving light pipes (Figure 15a). A laser beam is directed to the cantilever while the photodetector at the other end monitors the light intensity. 

The cantilever vibration is seen as the variation of light intensity by the photodetector. Low quality factors of $Q_{10}$~9–14 at 10 dB below peak vibration and resonant frequencies of ~300–7000 Hz have been measured at 70 dB SPL (Figure 15b) with linear dynamic range of 80 dB for sound input from 35 dB to 115 dB SPL. Here, we have seen that the array of cantilevers filters sound waves mechanically in parallel into different frequency bands. Compared to a microphone’s diaphragm, the array system may eliminate the function of analog-to-digital converter and digital signal processing [75]. The mechanical bank of cantilevers performing sound filtering mimics the function of BM. In Figure 15c, the apex of BM detects the lowest frequency and along the membrane’s length, the detected frequency increases from apex to base. The number of frequency points along the membrane represents the number of output channel. Figure 15d demonstrates the calculated cantilever lengths required to cover the frequency points on BM, mimicking the tonotopic mapping of frequency in BM. The resonator array can be designed to operate within the audible frequency range of 20 Hz–20 kHz. The geometrical dimension, shape and composition of the transducer influence the working frequency and bandwidth. For a simple cantilever, the mechanical resonant frequency $f_{c}$ is given by:

(1) $$f_{c}=\frac{\gamma^{2}}{2\pi }\;\sqrt{\frac{EI}{\rho wtl^{4}}}$$

where γ is 1.875 for fundamental mode vibration, E is the Young’s modulus, t represents thickness, w is the width, l is the length, I is the moment of inertia and ρ is the material’s density [77]. At resonant frequency, the cantilever will vibrate at a maximum amplitude. The quality factor $Q_{3}$ at 3 dB below peak vibration is defined by:

(2) $$Q_{3}=\frac{k}{2\pi f_{c}b}$$

where k is the elastic restoring coefficient and b is the damping coefficient of the cantilever structure.

**Figure 16:**
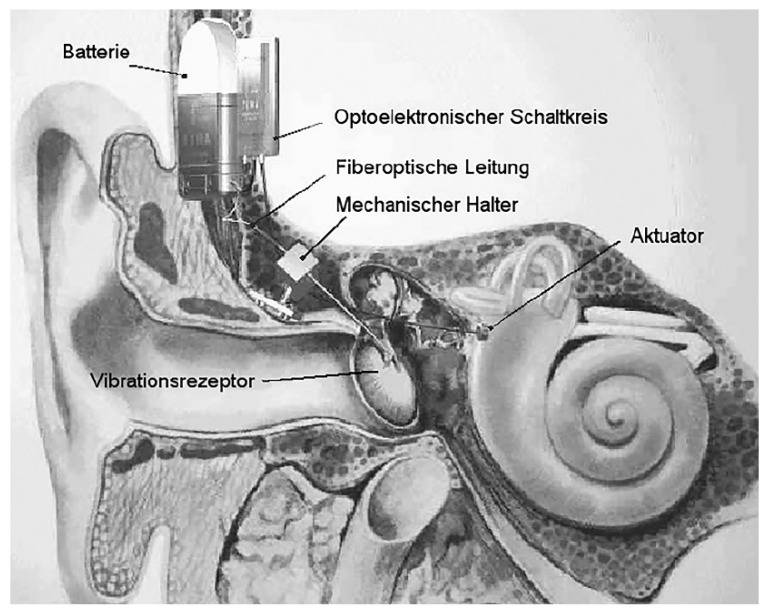
The proposed fibre-optic vibrometer system for middle ear implant. © 2002 IEEE. Reprinted, with permission, from [78].

Another optical-based microphone with a signal processing module is proposed in [78]. Vujanic et al. suggest a fibre-optic vibrometer as an implantable microphone in the middle ear for totally implantable hearing device. The device is simply an integration of the micro-optic components and fibre-optic signal lines with 0.125 mm diameter of the optical fibre. In this contactless detection, a laser beam is directed to the tympanic membrane or the ossicles via an elastic optical fibre. The structure vibration causes phase shift of the reflected light waves. Two photodiodes capture both the reflected and reference waves and transform them into electrical signals. The signals are processed by a signal processing circuit and the decoded information signals are sent to the cochlea. Macroscopic laboratory-built system shows the functional device and the miniaturised fibre-optic vibrometer could be used to drive the floating mass transducer in the middle ear implant system (Figure 16).

#### 3.1.2. Triboelectric-Based Sensor

**Figure 17:**
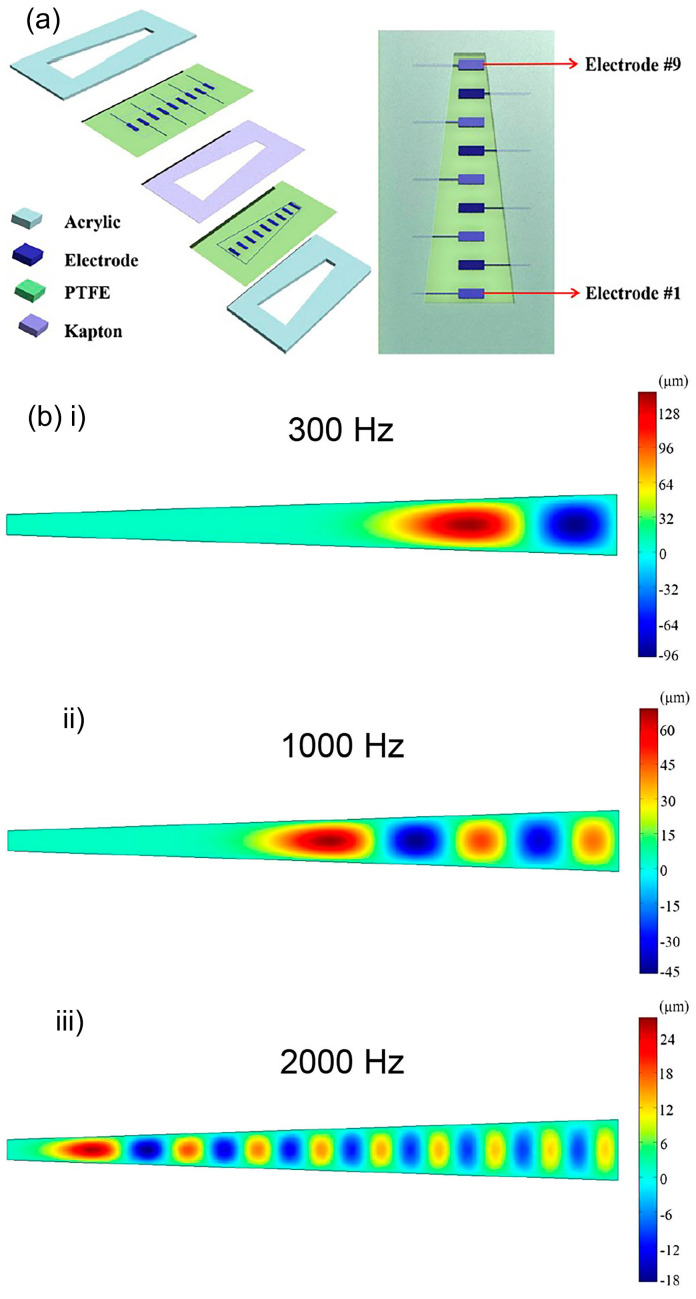
(**a**) The structural design of bionic acoustic triboelectric-based sensor. Two rectangular acrylic plates with trapezoidal cavity are used as the frames to support the PTFE membranes. The silver electrodes are numbered from electrode #1 to #9. (**b**) The simulated vibration characteristics of a trapezoidal PTFE membrane at 300 Hz, 1000 Hz and 2000 Hz of frequency input [76].

Triboelectric acoustic sensor performs acoustic-to-electrical transduction via contact electrification and electrostatic transduction mechanisms. A novel bionic cochlear basilar membrane acoustic sensor in conjugate with triboelectric nanogenerator is described in [76]. Two layers of trapezoidal polytetrafluoroethylene (PTFE) membrane with silver electrode arrays are separated by a Kapton polyimide (PI) film (Figure 17a). A narrow gap between the two PTFE membrane layers is created due to the placement of Kapton polyimide film in between.

The Kapton film’s thickness will determine the sound pressure detection limit and both Kapton film and PTFE membranes are strain-free. The trapezoid-shaped membrane is quite big with 10–30 mm width and 20 µm thickness. The acoustic vibration of PTFE membranes induces electricity generation. The triboelectric voltage output demonstrates a linear relationship with sound pressure. The device works within 20 Hz to 3000 Hz of frequency range with maximum vibration displacement of ~150 µm and maximum generated triboelectrical voltage of ~300 mV. A highly frequency selective function is achieved from the trapezoid-shaped membrane (Figure 17b). The large geometrical dimensions of the trapezoidal PTFE membranes produce large vibration displacements and thus, generate high triboelectric voltage. Also, the device is self-powered via the absorption of vibration energy from sound.

**Figure 18:**
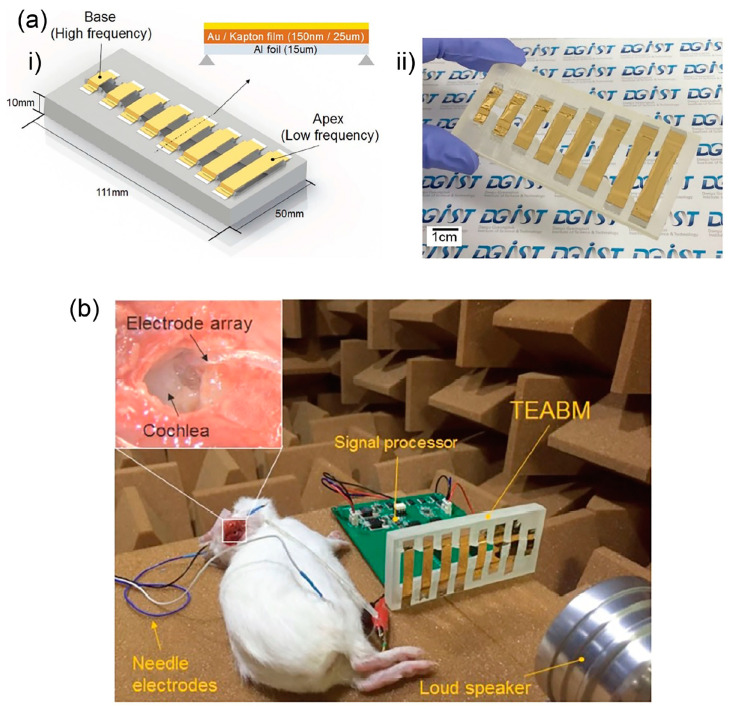
(**a**) (i) The schematic drawing and (ii) the optical image of eight Al/Kapton/Au beams on substrate. (**b**) The experimental setup for animal testing using the triboelectric acoustic sensor including the signal processor and the custom-made intra-cochlear electrode array [79].

Another triboelectric-based device by Jang et al. uses beam array of length 8.2–32 mm and width 6–8 mm [79]. In Figure 18a, each beam is stacked with aluminium foil as the bottom electrode and Kapton film with gold deposition as the top electrode. The two films are clamped in the ends to form a beam structure. Electrical output was generated during vibration via triboelectrification effect between Kapton film and aluminium foil with a sensitivity range of 1.74 mV/Pa to 13.1 mV/Pa and within the frequency range of 294.8 Hz to 2311 Hz. The tonotopic characteristics were also measured using an animal model (Figure 18b). The generated electrical signals from the triboelectric acoustic sensor were transformed into stimulating pulse signals via a signal processor. The applied pulse signals to the deafened guinea pigs via intra-cochlear electrode array manage to elicit the electrically evoked auditory brainstem response (eABR).

#### 3.1.3. Electromagnetic-Based Sensor

**Figure 19:**
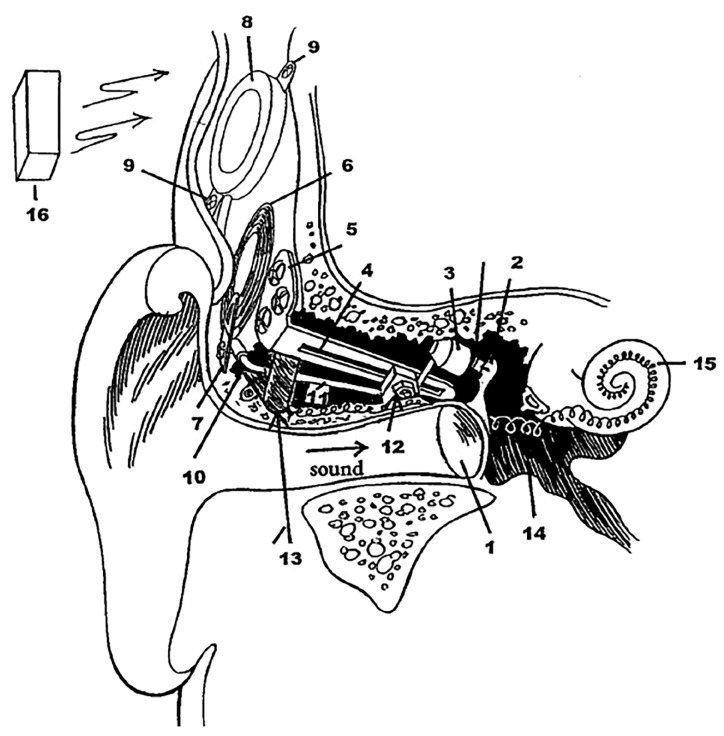
The neodymium iron boron magnet (2) at malleus is moved by the tympanic membrane (1) and interacts with the electromagnetic coil of 1900 turns (3). The supporting titanium shaft (4) is screwed to the temporal bone (5). The generated electrical signals from the coil are sent to the CI multichannel electrode array (15) in the cochlea. Reprinted from [80], Copyright (2001), with permission from Elsevier.

An electromagnetic displacement sensor is developed as an implantable middle ear sensor for a fully implanted cochlea implant [80]. The neodymium-iron-boron (NdFeB) magnet is attached onto one of the ossicle bones, malleus while the electric coil is positioned with a distance of 0.5–1 mm from the magnet (Figure 19). In the system, the incus bone is removed and the sensor transforms the sound-activated mechanical vibration from the tympanic membrane-ossicular chain into electrical signals through the electromagnetic induction between coil and magnet. The generated signals in the coil are amplified and transmitted to the speech processor before being sent to the cochlea via the stimulating electrode array. Flat frequency response within 5 dB is obtained from 200 Hz to 8000 Hz. The bench model test demonstrates low sensitivity of −30 dB at 3 kHz. A reduction in bandwidth is observed after implantation due to the load effect of the magnet that reduces the natural frequency of the ossicular chain. The power consumption is over 1 mW.

#### 3.1.4. Electrostatic-Based Sensor

**Figure 20:**
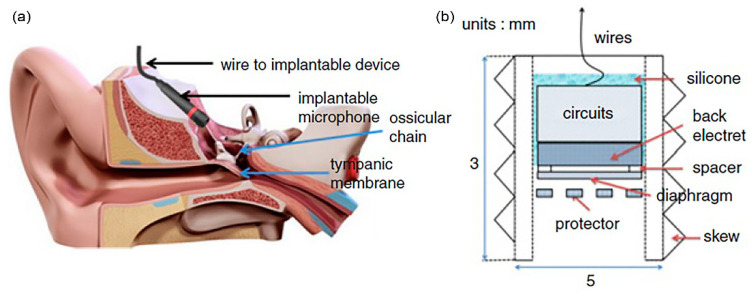
(**a**) The position of the implantable electrostatic-based microphone. (**b**) The block diagram of the microphone [81].

The electrostatic-based microphone uses a capacitance effect. In [81], a capacitive microphone is proposed to be implemented in the middle ear that measures the pressure variation inside the middle ear cavity, activated by the tympanic membrane vibration (Figure 20a). The microphone is directly mounted in a drilled tiny hole in the middle ear cavity. The ear canal and pinna effect helps to minimise loud noise signal that usually occurs in the implantable microphone inserted under the skin due to skin movement. The device installation requires simple surgery as the microphone is not attached or clipped onto the fragile and small ossicular chain. Figure 20b shows the electret condenser capacitive microphone in a screw-shaped titanium package with the membrane diaphragm made of titanium or stainless steel. The membrane diaphragm will vibrate according to the applied sound input signal whereas the rigid back plate is fixed, oppositely located. 10–15 dB attenuation was observed at high frequencies while no significant attenuation is measured from the low frequencies. The measured transmission loss is only 2.3 dB at 2 kHz.

**Figure 21:**
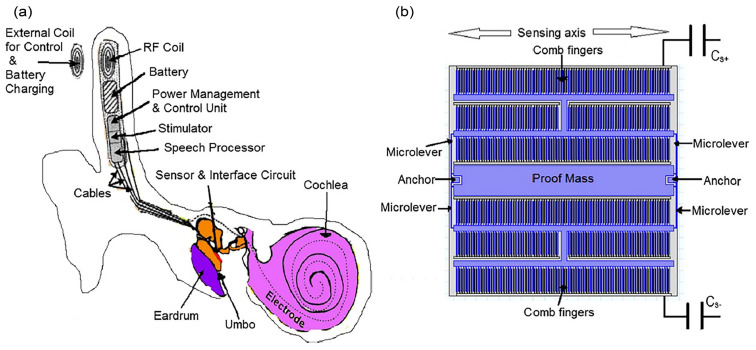
(**a**) The proposed fully implantable microphone at umbo in the middle ear. (**b**) The 2D layout of the accelerometer with differential capacitances. The capacitive sensed-fingers are connected to the proof mass through a system of microlevers [82]. Reprinted by permission from Springer Nature Customer Service Centre GmbH: Springer Nature MICROSYSTEM TECHNOLOGIES Numerical simulation and modelling of a novel MEMS capacitive accelerometer based microphone for fully implantable hearing aid, A. Dwivedi et al., COPYRIGHT (2019).

A novel capacitive accelerometer-based microphone is designed by Dwivedi et al. for a fully implantable hearing application [82]. The microphone can be surgically implanted in the middle ear bone structure, specifically at umbo (Figure 21a). The accelerometer can naturally pick up sound from umbo without signal distortion or attenuation. The accelerometer is proposed to be made of silicon with microlevers suspension. Other than providing the suspension for the proof mass, the microlevers serve as the mechanical amplifier. In Figure 21b, the accelerometer consists of a proof mass, four symmetrically arranged microlevers and two comb-finger stages for capacitive readout. The microlevers enhance the device sensitivity by enlarging the displacement of the proof mass, resulting in a larger displacement of the capacitive sense fingers and thus a higher capacitance signal. The incoming sound causes the eardrum and umbo to vibrate and exert an acceleration on the sensor along its sensing axis. The proof mass moves along the same axis through the microlevers that amplify its displacement. The output of each pair of microlevers is coupled to a comb-finger stage, where the displacement is converted into capacitance change. The device size of 1 mm^2^ achieves a nominal capacitive sensitivity of 5.91 fF/g and voltage sensitivity of 11.2 mV/g. Numerical simulation shows that the working frequency range of the microphone is between 100 Hz to 10 kHz with a flat response.

**Figure 22:**
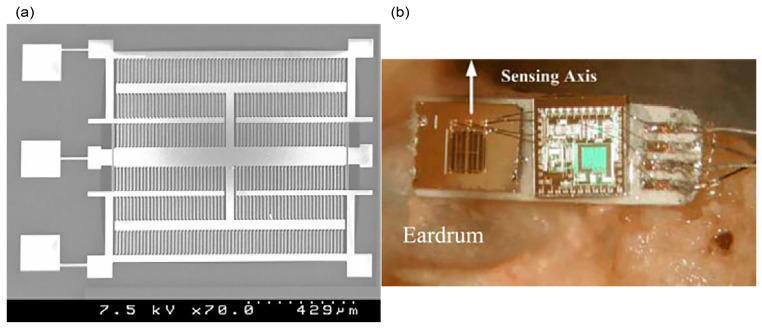
(**a**) The micrograph image of the fabricated capacitive accelerometer-based microphone. (**b**) The accelerometer is interfaced with the low-noise differential capacitance-to-voltage conversion circuitry and then implanted onto umbo. © 2012 IEEE. Reprinted, with permission, from [83].

A quite similar capacitive accelerometer-based microphone attached to umbo is fabricated by Young et al. [83]. The device is intended for middle ear hearing devices and fully implantable cochlear prosthesis in the future. In Figure 22a, the sensor is laid out in a symmetric arrangement with 189 sets of sensing fingers on each side of the proof mass, occupying 1 mm^2^ size. The proof mass is suspended by mechanical springs and the resonant frequency of the accelerometer is designed to be at 10 kHz. The implanted accelerometer on umbo is interfaced with low-noise electronic circuits on thin flexible substrate to convert the measured capacitance to voltage (Figure 22b). The device achieves a nominal capacitance of 2.4 pF. The attached sensor at umbo manage to detect a sound pressure level of 35–60 dB SPL at 500 Hz–8 kHz. The resonant frequency was measured at 6.44 kHz, lower than its designed value of 10 kHz due to over-etching of the mechanical suspension beam width. Improvements are necessary to interface the accelerometer-based microphone with the modern CI that demands sound detection level of below 45 dB SPL and frequency components of smaller than 300 Hz. 

Most of the commercially available microphones on the market are capacitive electret condenser microphones, which feature a rigid backplate and a flexible membrane diaphragm that deflects out of the wafer plane. The condenser microphone is essentially a capacitor that transduces sound into an electrical signal by varying the distance between two parallel plates of the capacitor, where the sound moves one of the plates i.e., the diaphragm. The electret condenser microphone eliminates the need of voltage across the plates as the material used in one of the plates is permanently charged. Hitherto, no implantable microphone sensor can come close to the excellent performance of the electret condenser microphones. Still, the capacitive-based microphones are not biocompatible and not small enough to be implanted. The reduction in size will give rise certain issues as the device sensitivity depends on the capacitance per unit area. Additionally, the capacitive sensors are highly susceptible to parasitic capacitance and nonlinearity behaviour. 

### 3.2. Piezoelectric-Based Mechanical Energy Sensor

**Figure 23:**
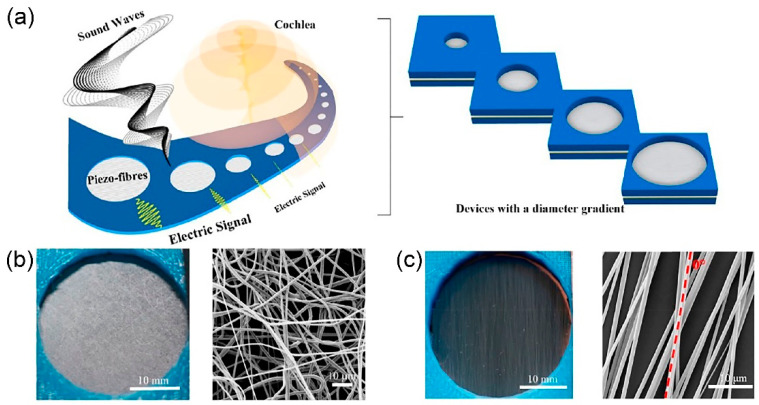
(**a**) The circular membranes of different diameters are fabricated using random and aligned P(VDF-TrFE) nanofibers, mimicking the function of a basilar membrane. The fibrous microphone membrane is based on electrospun piezoelectric polymer nanofibres with (**b**) random P(VDF-TrFE) fibres and (**c**) aligned P(VDF-TrFE) fibres [84]. Further permissions related to the material excerpted should be directed to the ACS.

Different types of piezoelectric materials, predominantly the inorganic piezoelectric/polymers, have been used to construct the implantable microphone for fully implanted hearing device system. Viola et al. fabricated an array of P(VDF-TrFE) circular membranes with diameter gradient for acoustic sensing [84]. In Figure 23a, the membrane array exhibits frequency selectivity behaviour that represents the tonotopy characteristics in cochlea. The circular membranes are fabricated using electrospinning method, producing random and aligned P(VDF-TrFE) nanofibres sandwiched in between copper electrodes (Figure 23b). Compared to thin film, the fibrous nanostructure is believed to provide more flexibility and sensitivity in the membrane. The fibre orientation and crystallinity can be tuned to alter the piezoelectric properties and the mechanical compliance of the membrane and thus designing the frequency selectivity and sensitivity of the microphone. Low frequencies of 100–400 Hz have been measured from the membrane array and the frequency response varies with respect to the changes in the membrane’s diameter and the electrospun fibres microstructural morphology. They have measured the increase of resonant frequency and the corresponding decrease in displacement and output voltage as the membrane’s diameter decreases. The random electrospun P(VDF-TrFE) circular membrane vibrates at lower resonant frequency with higher displacement and produces higher voltage sensitivity compared to the aligned nanofibres. The P(VDF-TrFE) acoustic sensor can generate electrical signals up to 17 mV.

**Figure 24:**
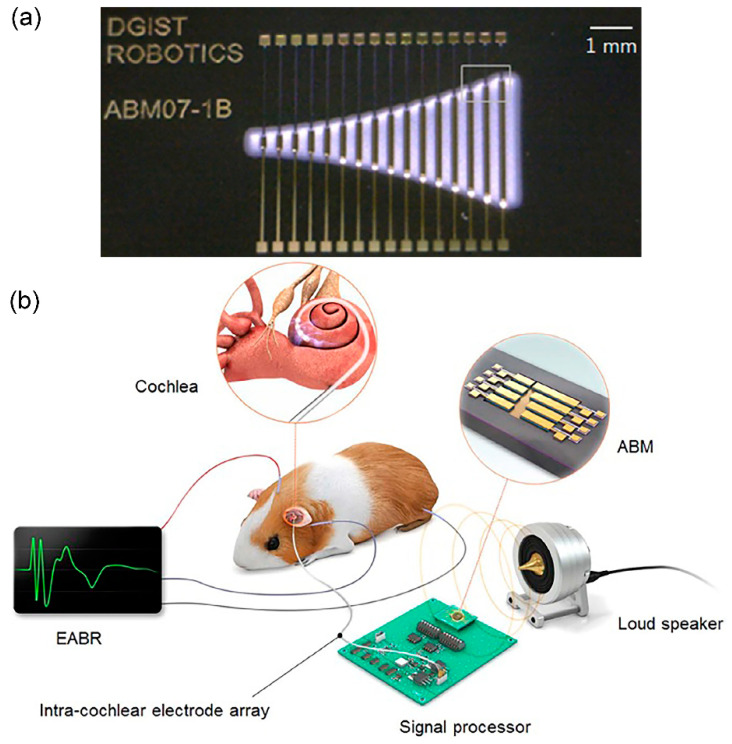
(**a**) The optical image of the fabricated Mo/AlN/Au beam array [86]. © IOP Publishing. Reproduced with permission. All rights reserved. (**b**) The schematic view of the experimental setup using Mo/AlN/Au cantilever array that generates piezoelectric voltage to the signal processor and intra-cochlear electrode array. The stimulating electrical signals onto the cochlea elicit eABR response from a deafened guinea pig [87].

In [85], Jang et al. report on a piezoelectric beam array made of aluminium nitride (AlN) to mimic the frequency selectivity of the basilar membrane and perform the acoustic-to-electrical energy conversion. Ten Mo/AlN/Au beams of length 1140–3300 µm and width 400 µm resonate at 10–37 kHz and produce sensitivity of 0.114–0.48 mV/Pa with the applied periodic chirped signals of 109.7 dB SPL. The device outputs in tens of µV are used to generate the pulse-width modulated signals that produce electrical stimulus pulses for the auditory nerves. In electrical polarisation matrix, the piezoelectrical signal in the direction of $D_{3}$ is given by:

(3) $$D_{31}=d_{31}T_{1}$$

where $d_{31}$ represents the piezoelectric coefficient and $T_{1}$ is the stress ensuing from the acoustic input signal. For a piezoelectric layer in between two electrodes, the generated electric field is in the perpendicular direction of the applied strain. The generated piezoelectric voltage $V_{piezo}$ is given by:

(4) $$V_{piezo}=\frac{\;d_{3}t_{piezo}}{\varepsilon^{T}}$$

where $\varepsilon^{T}$ is the electrical permittivity matrix and $t_{piezo}$ is the thin film piezoelectric layer’s thickness. A piezoelectric voltage of 340 µV was generated from one beam at 84.9 dB and increased to 957 µV, 1.85 mV and 3.55 mV at 97.6 dB, 105.4 dB and 112.4 dB, respectively. In [86], similar Mo/AlN/Au beams have been fabricated (Figure 24a) with length of 305–3200 µm and width of 200 µm giving a resonance frequency range of 2–36 kHz. They have suggested the use of piezoelectric polymer material for fabricating a less-stiff transducer structure in order to reduce the resonance frequency range. The developed residual stress during the fabrication of beam may cause the arc-shaped lateral deformation of the structure that affects the vibrational response of the beam.

In [87], the Mo/AlN/Au piezoelectric transducer array is changed from beams to cantilevers with the length range of 600–1350 µm. The sensitivity has been measured to improve remarkably to 0.354–1.67 mV/Pa. Lower resonance frequency range of 2.92–12.6 kHz is attained at 101.7 dB SPL (42 Hz–20 kHz). Using cantilevers, the transducers manage to work at lower frequency range with better sensitivity and are smaller in size compared to beams. In Figure 24b, an animal model is used where the device output is digitally processed to become the electrical stimulus for the auditory neurons. The stimulus is delivered to the cochlea of the deafened guinea pigs to initiate eABR using the implanted intra-cochlear electrode array. The measured eABR increases as the applied acoustic stimuli increases from 75 dB SPL to 95 dB SPL. The larger the piezoelectric voltage, the wider the pulse width, and thus more charges can be applied onto the intra-cochlear electrode array. In [88], the piezoelectric Mo/AlN/Au beam array has been employed to mimic BM’s function in fluid mediums. In fluid, the vibration displacements and resonant frequencies decrease ~4 times and ~3.6 times, respectively, due to high damping effect.

**Figure 25:**
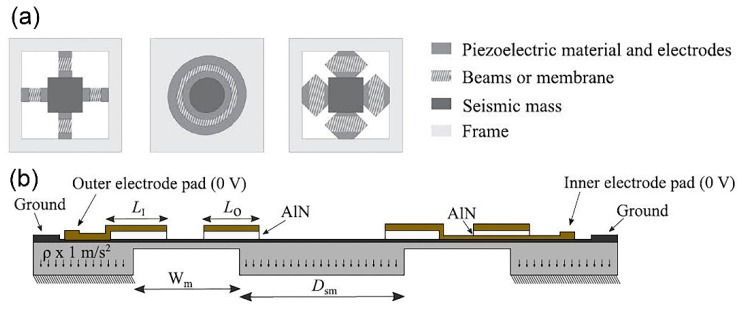
(**a**) Top view of the trampoline (left), annular (middle) and hexagonal beams with square seismic mass (right) piezoelectric accelerometers. (**b**) The geometric parameters, boundary conditions and body force imposed in the finite element model, represented in a sectional view of the annular accelerometer [89].

Gesing et al. have modelled and fabricated a piezoelectric accelerometer-based implantable microphone coupled to the ossicular chain for hearing devices [89]. AlN has been chosen as the piezoelectric material and different design of annular, trampoline and hexagonal beams with square seismic mass configurations have been considered for the accelerometer (Figure 25). The best design was found to be 2 × 2 mm^2^ annular accelerometer with 500 nm AlN thickness. High net charge sensitivity is achieved within the working frequency range of 100 Hz–10 kHz with the resonant frequency of the microphone measured at 19.1 kHz. The accelerometer performance agrees well with its finite element model prediction. The device can detect sound pressure level only above 60 dB between 600 Hz to 10 kHz.

**Figure 26:**
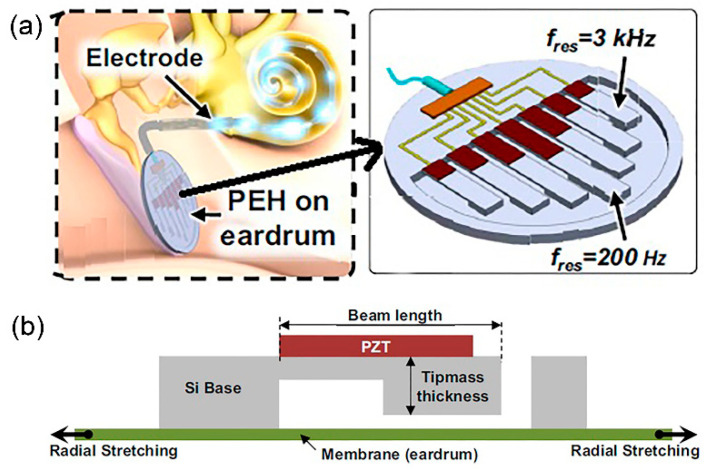
(**a**) The proposed piezoelectric cantilevers placement on the tympanic membrane (**b**) the bulk piezoelectric cantilever on the eardrum membrane model. © 2013 IEEE. Reprinted, with permission, from [90].

In Figure 26a, cantilevers are proposed to be implanted on the tympanic membrane to stimulate the auditory nerves in cochlea [90]. The stimulating electrode array, similar to the ones in the conventional CIs, can be used to transmit the generated piezoelectric voltage from the cantilevers to the auditory nerves. In Figure 26b, a silicon bulk cantilever of 4.25 mm length, 4 mm width and 20 µm thickness with a tip mass and Au/PZT/Au piezoelectric layer on top has been fabricated. The device is placed on a simulated eardrum membrane model where its first natural frequency is expected to be at 550 Hz. From measurement, the resonant frequency is measured to be 474 Hz with the generated RMS voltage and power of 588 mV and 1.33 µW, respectively, at an acceleration of 0.1 g. The increase of acceleration level to 1.6g increases the output power to 137.5 µW. The device can generate adequate signals to stimulate cochlea at typical eardrum vibrations.

**Figure 27:**
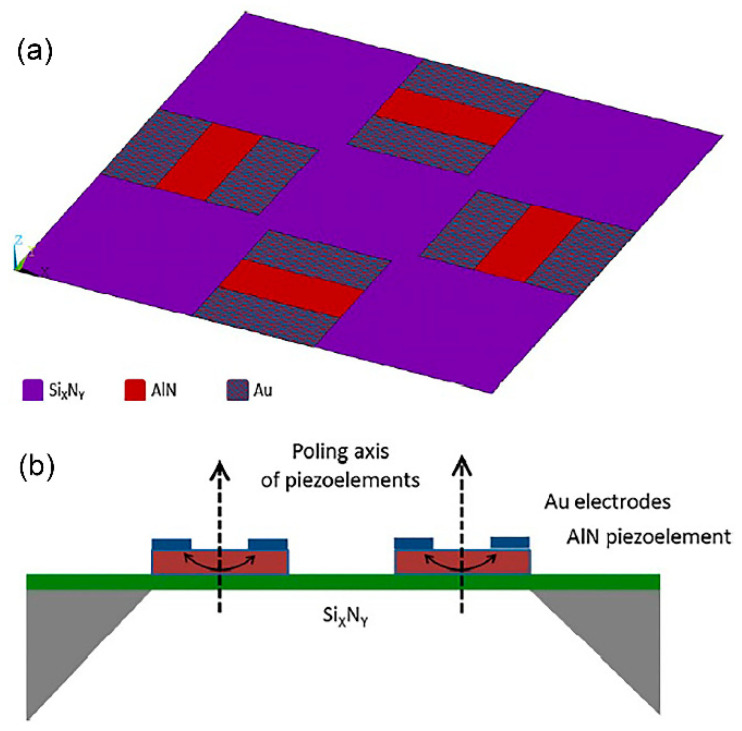
(**a**) Si_x_N_y_ membrane of dimension 0.5 mm × 0.5 mm with four AlN/Au piezoelectric elements. (**b**) The cross-section schematic of device showing AlN and gold electrodes operating in mode 33. Reprinted from [43], Copyright (2015), with permission from Elsevier.

Žák et al. proposed an array of 24 isolated membranes with different dimensions to detect and filter acoustic signals [43]. The freestanding membrane is made of Si_x_N_y_ with four AlN piezoelectric elements and gold electrodes placed on top edge of the square Si_x_N_y_ membrane (Figure 27a). The AlN layer has poling axis in orthogonal direction and the layer operates in piezoelectric mode 33, like IDEs (Figure 27b). Membrane vibration from acoustic waves actuation bends the AlN layer and the induced mechanical strain in AlN generates electrical potentials between the gold electrodes. The maximum amplitude of the generated voltage is simulated to be 0.38 V on the gold electrodes. The membrane array integrates with the signal processing electronics, stimulation nerves electrodes and energy harvesting system by which each membrane represents one frequency channel with its own electronics for sensing, processing and electrode nerves excitation. The total power consumption per each channel is approximately 10 µW at 2 V of power supply. Žák et al. concluded that the use of membrane array is more suitable for sensing ambient acoustic pressure compared to cantilever array.

**Figure 28:**
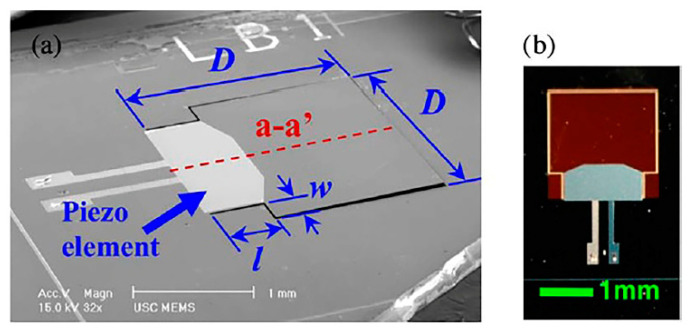
(**a**) Scanning electron microscopy image and (**b**) top view optical image of a single paddle-shaped cantilever microphone. © 2013 IEEE. Reprinted, with permission, from [91].

An array of thirteen unimorph piezoelectric monocrystalline silicon microphones has been fabricated for acoustic sensing [91]. The cantilever-type diaphragm topology of paddle-shaped transducers are developed with Al/ZnO/Al piezoelectric layer on top (Figure 28). Paddle shape transducer can give lower resonant frequency compared to the typical rectangular cantilever. They used the stress-compensating layer and the stress-free monocrystalline silicon diaphragm to minimise the influence of residual stress on the fabricated microphone structure. The microphone array can operate in a broadband mode where the overlapping resonance boosts the overall sensitivity of the array. The maximum resonance sensitivity has been measured to be 10.8–202.6 mV/Pa from 860 Hz to 6263 Hz. 

An array of Pt/AlN/Pt/AlN/Pt bimorph piezoelectric intracochlear acoustic transducers is implanted inside a living guinea pig cochlea for acoustic pressure sensing [92]. Four piezoelectric cantilevers of 400 µm wide and varying length that spans from 300 µm to 443 µm were coated with 50 nm alumina and 2 µm parylene to enhance the device durability. Fluid-loaded resonance frequencies for the cantilevers outside the ear are measured in a range of 5.6–13.5 kHz whereas the in vivo piezoelectric voltage of 1.3–79.7 µV are measured in response to 80 dB SPL-95 dB SPL of input stimuli over frequencies of 1–14 kHz. The device is capable of reading out the voltage in a living cochlea driven by the external acoustic excitation. The sensitivity measured in a living cochlea falls in the range of 1.2–71 µV/Pa while from a benchtop experiment, the sensitivity ranges from 0.05–12.6 µV/Pa. This demonstrates that the intra-cochlear pressure signals are higher than those outside the ears and those reaching the subcutaneous microphones. Intra-cochlea acoustic sensors have the potential to improve the existing fully implanted CIs by replacing the external or subcutaneous microphones of the auditory prosthetics.

Five bimorph piezoelectric cantilevers of 400 µm wide, 3.2 µm thick and 150–210 µm long are designed to resonate in fluid at 20 kHz to 40 kHz [93]. The AlN cantilevers with platinum electrodes are fabricated to suspend on a silicon backbone support. The device is implanted in the straight section of the scala tympani of a guinea pig where the cantilever locations correspond to the tonotopic points. Through direct piezoelectric effect, the motion in perilymph deflects the cantilever which produces a potential across the cantilever’s outer electrodes. The device converts the mechanical vibration of the perilymph into electrical current that is emitted back to the perilymph fluid. This device however is not capable of producing sufficient current to evoke eABR. In air, the actuated transducers are measured at 80.3–134.2 kHz with a quality factor Q~170 while in water the resonance frequency range shifted to 24.3–41 kHz with Q~5.8.

Jung et al. fabricated multichannel piezoelectric acoustic sensor that performs frequency filtering and mechanoelectrical transduction without external energy source and signal processing unit [94]. The maximum piezoelectric constant of the poled Au/PVDF/Au thin film trapezoidal membrane layer is measured to be 4.05 pC/N. The device is tested with a mouth simulator. The trapezoidal piezoelectric thin film membrane with width varies from 1 mm to 8 mm demonstrates the tonotopy characteristics of the basilar membrane by filtering sound within the frequency range of 2.5–13.5 kHz. The maximum piezoelectric output upon acoustic signal input of 94 dB SPL is 6.3 mV peak to peak. 

A piezoelectric artificial cochlea is made of 40 µm thick PVDF membrane fixed on a stainless-steel substrate with a trapezoidal slit [95]. The width of the membrane is linearly varied from 2.0 mm to 4.0 mm. A detecting aluminium electrode array with 24-elements of 0.50 mm × 1.0 mm rectangles are located in a centre line of longitudinal direction on the PVDF membrane. The electrodes with 100 nm thickness compared to 40 µm PVDF membrane are assumed to not strongly affect the membrane’s vibration behaviour. The ground point is the common aluminium electrode for all discrete electrodes on the lower side of the membrane. The basic characteristics in terms of vibration and acoustic-to-electric conversion are investigated both in the air and in the silicone oil which is a model of lymph fluid in cochlea. Due to some constrictions during measurement, the silicone oil is only placed at one side, below the membrane. The membrane’s resonant frequency has been measured from 6.6 kHz to 19.8 kHz in the air and from 1.4 kHz to 4.9 kHz in the silicone oil, respectively. The effect of fluid-structure (acoustic wave-membrane) interaction increases the effective mass for vibration and thus causes the decrease in resonant frequency performance in the silicone oil. It was found that viscous fluid suppresses standing waves and improves frequency selectivity. The amplitude of piezoelectrical signal is ~16 µV at 90 dB SPL in silicone oil. The electric output needs to be amplified for effective nerve cells stimulation or the membrane can be made thinner for larger voltage generation. The fabricated device was relatively too big to be implanted.

Yip et al. mounted a PZT sensor at the umbo of the malleus within the middle ear to detect sound-induced motion from the ossicular chain and convert the mechanical motion into electrical signals [96]. The generated charges are converted to output voltage via a charge amplifier. The implantable acoustic sensor device is interfaced with a system-on-chip (SoC) that also integrates the sound processor and neural stimulator, all in one chip measuring 3.6 mm × 3.6 mm. The sensor’s readout is amplified and fed into the sound processor and neural stimulator to stimulate the cochlea directly. The piezoelectric sensor is implanted and measured from a human cadaveric ear. The device operates within 200 Hz to 10 kHz and detects sound over 50 dB dynamic range from 40 dB SPL to 90 dB SPL. Eight stimulating electrodes provide adequate spectral resolution while minimising the hardware complexity. The SoC in 8-channel mode consumes a total power of 572 μW. The piezoelectric sensor, analogue to digital converter and sound processor consume only 2% of the total power, while 98% of the power is consumed by the stimulator circuits. Therefore, stimulation power saving from the mixed-signal arbitrary waveform neural stimulator is necessary to deliver the energy-optimal stimulation pulses to the auditory nerves. 

Envoy Esteem by Envoy Medical Corporation is a fully implanted middle ear hearing system. It consists of an implantable piezoelectric sensor, sound processor, driver and battery [17]. The sound processor and battery are placed in a subcutaneous pocket posterior to the mastoid cavity while the driver and sensor are positioned in the mastoid cavity. The implantable piezoelectric sensor acts as an internal microphone. The tip of the sensor is in contact with the malleus whereas the sensor itself is attached to the incus. As the tympanic membrane moves, the vibrations of the ossicular chain are detected by the piezoelectric microphone sensor, which in turn generates a voltage proportional to malleus vibrations. The voltage is routed to the sound processor via the insulated wires, where the signal is processed and amplified. The sound processor relayed the information to the driver which is a piezoelectric actuator, with its tip contacting the head of the stapes, resulting in its vibration. The mechanical motion of stapes stimulates the cochlea. Speech reception threshold is 29.4 dB and the microphone’s operating range is in between 125 Hz to 8 kHz. A summary of the discussed research work on the implantable sensor for hearing devices is tabulated in Table 4. 

### 3.3. The Future Aspects of Piezoelectric/Polymeric Implantable Microphone for Totally Implanted Hearing Device

Yip et al. have found that the main power consumption in their fabricated hearing device comes from the stimulator where it represents >90% of the total power with the microphone and sound processor consuming only in microwatts compared to a few miliwatts from the stimulator circuits [96]. The totally implanted device consumes 572 μW, 425 μW and 281 μW in 8-, 6- and 4-channel systems, respectively, demonstrating lower power consumption in systems with fewer channels. They insist that the effective number of the stimulating electrode array should be in balance between the speech recognition performance, the hardware complexity and power consumption. In a quiet condition, 3 to 4 channels of spectral information can already result in good speech recognition performance where the increase of channel number from 1 to 4 improves the average performance. However, no differences have been observed as the number of stimulation electrodes increases from 7 to 20. In a noisy environment, the improvement is only up to 7 stimulating electrodes where the users with low levels of speech recognition did not benefit from more than 4 electrodes. The effective number of spectral channels possible with eABR hearing is usually limited by the spreading between electrodes. 

The disadvantage of a commercialised standard microphone lies in its flat frequency response and thus it needs to combine with the high-performance digital speech processor to perform the desired frequency filtering [43]. A bank of selective filters created from an array of transducers or a trapezoidal membrane proposes filtering solution without a speech processor. The primary benefit would be that the digital resources are left free for other computationally intensive task as the transducer array reduces the work and size of the processor. Simultaneous filtering by the array also reduces latency in the output signals [75]. A typical CI that uses digital signal processor institutes latency in audio signals. Frequency selectivity in a trapezoidal membrane has been found to be poor due to the mechanical coupling of the membrane among the sensing electrode channels on the same membrane [87]. Thus, beam or cantilever arrays are preferable since the structures are separated from each other, giving clear frequency selectivity. 

Wider bandwidths of the transducers have been measured at the higher frequency region of the audible range, as the transducers’ size decreases. This results in lower resolution of the processed sound signals at high frequency, producing lower quality of frequency selectivity. Increasing the number of transducers in the array with smaller jumps in length helps to produce narrower bandwidth and covering more frequency points. In this approach however, a large number of stimulating electrodes is required to cater the large number of frequency points/channel outputs and there is a limit on how many electrodes that can be implanted in cochlea. High-density microelectrode array is suggested in [75] by adopting hairlike electrodes to the basilar membrane. There would be a high density of bond points due to the hundreds of electrical connections to be made. Bear in mind that the cochlea is able to separate incoming sound signals into ~3500 channels of frequency information [87]. It might be necessary to design a high-density transducer array for high quality hearing, although Yip et al. have reported otherwise [96].

Many of the reviewed transducers in the array design have the difficulty of working in the lower region of the audible range. The commonly used micromachining materials like silicon, ceramics and metals typically exhibit low damping and high Young’s modulus. These pose challenges in designing the transducers to work at low frequency region. Cantilevers can work at lower operating frequency range due to its fixed-free structure compared to the fixed-fixed beam. Other than reducing the structure’s stiffness, mass can be added to obtain lower frequency response at the same length. Putting a mass at the free end of a cantilever or at the central beam length would increase its effective mass and thus decrease the modal frequency. The integration of polymer materials that possess high damping and low Young’s modulus value with the piezoelectric transducers can result in less stiff structures that help to decrease the transducer’s operating frequency. 

For the accelerometer-based microphone implanted in the ossicular chain of the middle ear, there is a possibility for the device to be misaligned during the implant surgery procedure. It is bound to happen, especially on the curved umbo surface, causing deviation of the device from the desired sensing axis leading to performance degradation [97]. The commonly used inorganic piezoelectric materials are too stiff to make a good contact with umbo. In addition, the employment of an optical fibre approach is difficult since it is a complex system with the possibility of temporary signal loss. Lastly, the large weight, size and loading effect impede the attachment of electromagnetic-based microphones on the ossicular bones, and plus there is the incompatibility issue with MRI. These weaknesses give us an inkling of how the implementation of piezoelectric/polymeric nanocomposite or multilayer-based microphone might outweigh other material types due to its elastomeric and flexible nature. The polymer-based piezoelectric transducer with proper device attachment and high piezoelectric performance might be the fitting implantable microphone for the totally implanted hearing device system.

## 4. Conclusions

The mechanoelectrical micro-devices performance in sensing and energy harvesting for hearing systems have been reviewed closely. The study mainly focuses on the configuration structure, principle of operation, fabrication method, materials growth and deposition of polymer-based piezoelectric transducers towards the development of a totally implanted cochlear device. Mechanical vibration energy carried by sound is an energy source that is naturally available surrounding the hearing device. In vivo piezoelectric energy harvesting from organs’ motion have been investigated for the development of continuous and self-powered energy harvesters. Many advantages in utilising polymer-based piezoelectric materials have made the piezoelectric transduction the foremost mechanical energy harvesting mechanism. Different forms, such as thin film, nanofibres and embedded extruded rods of the inorganic, organic and composite piezoelectric materials have been discussed. For developing the implantable microphones, we have seen that the incorporation of the ear drum and middle ear bones with the microphone sensor is favoured for sound conduction as these structures can naturally pick up sound and process it. The piezoelectric, electrostatic, optical, triboelectric and electromagnetic-based mechanical energy sensors have been reviewed, and with the sensors’ structure may adopt the configuration of a microphone diaphragm, accelerometer, displacement sensor, transducer array or trapezoidal membrane. Unimorph and bimorph piezoelectric transducers have shown interesting outputs with the inorganic piezoelectric polymers surmount as the suitable material for an implantable microphone. The piezoelectric transducer array and trapezoidal-shaped membrane have the advantage of analogue frequency filtering that minimises the dependence of the hearing device microphone sensor on signal processing. In reviewing the literatures, it seems that the research work done on transducer array/trapezoidal membrane for hearing device application is still in its infancy and most of the studies are focused on understanding the fundamental aspects of the biomimicking process. On the contrary, the speech processor uses the well-established technology that has been implemented in the hearing systems for so long. Therefore, rather than just focusing on the implantable microphone and in vivo energy harvester, attention should also be given on making the speech processor smaller and implantable. The monolithic integration of the implantable microphone, in vivo energy harvester, energy storage, signal processing unit and stimulation circuits with the existing electrode array of a CI or FMT of a MEI might lead to the realisation of a fully implanted hearing device system.

## Figures and Tables

**Table 1:** Descriptions of the commercially available hearing systems.

Hearing System	Hearing Loss Condition	System Operation	Manufacturer
Cochlear implant (CI)	Severe to profound sensorineural	Bypass outer ear, middle ear and inner ear’s BM and hair cells. Direct stimulation of auditory nerves.	HiRes Ultra 3D by Advanced Bionics [14] Nucleus^®^Systems by Cochlear Americas [15]
Middle ear implant (MEI)	Mild to severe sensorineural, conductive or mixed	Bypass the outer ear. Transduce mechanical vibrations in the middle ear and transmit them to stimulate the cochlea.	Vibrant Soundbridge by MED-EL [16] Envoy Esteem by Envoy Medical [17]
Hearing aid	Mild to moderate sensorineural	Amplify and transmit sound to the cochlea via ear canal and middle ear structures.	Evoke by Widex [18] More by Oticon [19]
Bone -anchored hearing aid (BAHA)	Mild sensorineural, conductive or mixed	Bypass the outer ear and middle ear. Transmit sound to the cochlea via skull’s bones.	Baha^®^Attract by Cochlear Corp. [11] BONEBRIDGE by MED-EL [20]

**Table 2:** Summary of in vivo energy harvesters for implanted medical devices.

Energy Source	Transduction Mechanism	Device Application	Input Excitation	Output Performance
Mechanical	Piezoelectricity	Lead-free cardiac pacemaker	Heartbeat vibrations	10 µW [35,36]
Mechanical	Piezoelectricity	Cardiac pacemaker	Pacemaker’s lead/heart motion	0.6 Vpp [37]
Mechanical	Piezoelectricity	Cardiac sensor	porcine heart motion	17.8 V and 1.74 μA [38]
Mechanical	Piezoelectricity	Blood pressure sensor	aorta’s pulsating motion	10.3 V, 400 nA and 631 nW [39]
Heat	Thermoelectricity	Cardiac pacemaker	Temperature gradient between skin and body core	100 µW [28]
Mechanical	Piezoelectricity	Brain pacemaker	Muscles/organ motion	0.57 mA and 11 V [41]
Mechanical	Piezoelectricity	Brain pacemaker	Human mandible motion	1 Vpp [42]
Mechanical and heat	Electromagnetism and thermoelectricity	Cochlear implant	neck muscles/arteries motion (electromagnetism) and temperature gradient between skin and body core (thermoelectricity)	~1.4 Vpp and ~1.8 mW (electromagnetism) and 300 µW (thermoelectricity) [43]
Electrochemical gradient	Electrochemistry	Cochlear implant	Ionic concentration gradient in inner ear’s fluid	1.12 nW [44]

**Table 3:** Summary of piezoelectric material, potentially used for energy harvester in cochlear device application.

Material Category	Material Type	Formation Structure	Transducer Configuration	Implanted Location	Output Performance
Inorganic piezoelectric	PZT	Thin film	Au/PZT/Au on Si cantilever with a tip mass	Eardrum	1.5 mW/cm^3^ [46]
	PZT	Thin film	Pt/PZT/Pt on Si/Si_3_N_4_ cantilever with SU-8 top proof mass	-	23 nW, first mode [47]
	PZT	Thin film	Al/PZT/Pt on SiO_2_ diaphragm	-	23.8 µW/m^2^, 100 dB SPL [49]
	PZT	Thin film	Pt/PZT/Au on Si cantilever with a tip mass	Eardrum	391.9 mV/Pa, 900 Hz [50]
	ZnO	Thin film	Rectangular and perforated tapered ZnO/Si cantilever with Si proof mass	Eardrum	0.35 V (rectangular) [51] 0.75 V (perforated tapered)
Organic piezoelectric	PVDF	Thin film	Vertical PVDF cantilevers	Scala tympani	More than 1 mV [58]
	PVF_2_	Thin film	Ni/PVF_2_/Cu and PPY/PVF_2_/PPY cantilevers	-	0.15 mV/Pa, 125 dB SPL [59]
	PVDF	Thin film	Al/PVDF/Al membrane on a slit of stainless plate.	Scala tympani	16 µV, 90 dB SPL [60]
	P(VDF-TrFE)	Thin film	Pt/P(VDF-TrFE)/Au membrane on a silicon frame	Scala tympani	0.14 mV–5.88 mV, 100 dB SPL [60]
Piezoelectric/polymeric	BTNP/ PVDF	Nanoparticles/nanofibres	Thin meshes of BTNP/PVDF nanoparticles/nanofibres composite	Cochlear epithelial cells	1 mV/N [68]
	PZT/PET	Thin film/thin film	PET/PZT/Au on silicone-based membrane	Basilar membrane	59.7 µV, 40 dB SPL [69]
	PZT/PI	Nanoribbon/thin film	Pt/PZT/Au on PI layer	Bovine heart	1.2 µW/cm^2^, 5 layers [70]
	PMN-PT/PET	Thin film/thin film	Au/PMN-PT/Au on PET layer	Heart	8.2 V [71]
	PZT/ epoxy	Nanorods/ thin film	Extruded PZT rods in epoxy matrix nanocomposite	-	~100 pC/N [73]
	PZT/PU	Nanorods/foamed thin film	Extruded PZT rods in foamed PU matrix nanocomposite	-	225 pC/N [74]

**Table 4:** Implantable sensor for totally implanted cochlear devices.

Transduction Mechanism	Material Structure	Implantable Sensor Configuration	Implanted Location	Output Performance
Optical	SU-8 thin film	Cantilever microphone array	-	$f_{c}$$\sim 300–7000\;\mathrm{Hz},\;80\;\mathrm{dB},\;Q_{10}$~9–14 [75]
	Optical fibre	Optical vibrometer	Middle ear	$f_{c}$~5 kHz, 85 mVpp [78]
Triboelectric	PTFE/PI/PTFE thin film	Trapezoidal membrane with silver electrodes	-	$f_{c}$~20–3000 Hz, 300 mV [76]
	Al/Kapton/Au thin film	Beam microphone array	-	$f_{c}$~294.8–2311 Hz, 1.74–13.1 mV/Pa [79]
Electromagnetism	NdFeB magnet	Magnet and electric coil displacement sensor	Middle ear	$f_{c}$~200 Hz–8 kHz, 5 dB, 1 mW [80]
Electrostatic	Ti or steel thin film	Electret condenser microphone membrane diaphragm	Middle ear	$f_{c}$~200 Hz–10 kHz, 2.3 dB transmission loss [81]
	Si thin film	Proof mass with microlevers and comb fingers accelerometer	Middle ear	$f_{c}$~100 Hz–10 kHz, 11.2 mV/g [82]
	Si thin film	Proof mass with springs and comb fingers accelerometer	Middle ear	$f_{c}$~500 Hz–8 kHz, 35–60 dB SPL [83]
Piezoelectricity	Cu/P(VDF-TrFE)/Cu nanofibres	Circular membrane diaphragm microphone array	Basilar membrane	$f_{c}$~100–400 Hz, 17 mV [84]
	Mo/AlN/Au thin film	Beam microphone array	Basilar membrane	$f_{c}$~10–37 kHz, 0.114–0.48 mV/Pa [85]
	Mo/AlN/Au thin film	Cantilever microphone array	Basilar membrane	$f_{c}$~2.92–12.6 kHz, 0.354–1.67 mV/Pa [87]
	AlN	Annular, trampoline or hexagonal beams with square seismic mass accelerometer	Middle ear	$f_{c}$~600 Hz–10 kHz, above 60 dB [89]
	Au/PZT/Au thin film on Si	Si cantilevers with tip mass microphone array	Eardrum	$f_{c}$~474 Hz, 588 mV, 1.33 µW at 0.1g [90]
	AlN/Au thin film on Si_x_N_y_	Si_x_N_y_ square membrane microphone array	-	$f_{c}$~9724 Hz, 0.38 V [43]
	Al/ZnO/Al thin film on Si	Si paddled-shaped cantilever microphone array	-	$f_{c}$~860–6263 Hz, 10.8–202.6 mV/Pa [91]
	Pt/AlN/Pt/AlN/Pt bimorph thin film	Cantilever microphone array	Cochlea	$f_{c}$~1–14 kHz, 1.2–71 µV/Pa [92]
	Pt/AlN/Pt/AlN/Pt bimorph thin film	Cantilever microphone array	Scala tympani	$f_{c}$~24.3–41 kHz, Q~5.8 [93]
	Au/PVDF/Au thin film	Trapezoidal membrane microphone	-	$f_{c}$~2.5–13.5 kHz, 6.3 mVpp [94]
	Al/PVDF/Al thin film	Trapezoidal membrane microphone	-	$f_{c}$~1.4–4.9 kHz, 16 µV [95]
	PZT thin film	Cantilever force sensor	Eardrum	$f_{c}$~200 Hz–10 kHz, 50 dB [96]
	-	Microphone	Middle ear	$f_{c}$~125 Hz–8 kHz, threshold at 29.4 dB [17]

## Data Availability

Not applicable.
